# Co-trimoxazole or multivitamin multimineral supplement for post-discharge outcomes after severe anaemia in African children: a randomised controlled trial

**DOI:** 10.1016/S2214-109X(19)30345-6

**Published:** 2019-09-16

**Authors:** Kathryn Maitland, Peter Olupot-Olupot, Sarah Kiguli, George Chagaluka, Florence Alaroker, Robert O Opoka, Ayub Mpoya, Kevin Walsh, Charles Engoru, Julius Nteziyaremye, Machpherson Mallewa, Neil Kennedy, Margaret Nakuya, Cate Namayanja, Julianne Kayaga, Eva Nabawanuka, Tonny Sennyondo, Denis Aromut, Felistas Kumwenda, Cynthia Williams Musika, Margaret J Thomason, Imelda Bates, Michael Boele von Hensbroek, Jennifer A Evans, Sophie Uyoga, Thomas N Williams, Gary Frost, Elizabeth C George, Diana M Gibb, A Sarah Walker

**Affiliations:** a Department of Medicine, Imperial College London, London, UK; b Nutrition Research Section, Imperial College London, London, UK; c Busitema University Faculty of Health Sciences, Mbale Campus and Mbale Regional Referral Hospital Mbale, Mbale, Uganda; d Department of Paediatrics, Makerere University and Mulago Hospital, Kampala, Uganda; e Soroti Regional Referral Hospital, Soroti, Uganda; f Kenya Medical Research Institute–Wellcome Trust Research Programme, Kilifi, Kenya; g College of Medicine, and Malawi-Liverpool-Wellcome Trust Clinical Research Programme, Blantyre, Malawi; h School of Medicine, Dentistry and Biomedical Science, Queen's University, Belfast, UK; i Liverpool School of Tropical Medicine and Hygiene, Liverpool, UK; j Emma Children's Hospital, Academic Medical Center, Amsterdam, The Netherlands; k Department of Paediatrics, University Hospital of Wales, Cardiff, UK; l Medical Research Council Clinical Trials Unit at University College London

## Abstract

**Background:**

Severe anaemia is a leading cause of paediatric admission to hospital in Africa; post-discharge outcomes remain poor, with high 6-month mortality (8%) and re-admission (17%). We aimed to investigate post-discharge interventions that might improve outcomes.

**Methods:**

Within the two-stratum, open-label, multicentre, factorial randomised TRACT trial, children aged 2 months to 12 years with severe anaemia, defined as haemoglobin of less than 6 g/dL, at admission to hospital (three in Uganda, one in Malawi) were randomly assigned, using sequentially numbered envelopes linked to a second non-sequentially numbered set of allocations stratified by centre and severity, to enhanced nutritional supplementation with iron and folate-containing multivitamin multimineral supplements versus iron and folate alone at treatment doses (usual care), and to co-trimoxazole versus no co-trimoxazole. All interventions were administered orally and were given for 3 months after discharge from hospital. Separately reported randomisations investigated transfusion management. The primary outcome was 180-day mortality. All analyses were done in the intention-to-treat population; follow-up was 180 days. This trial is registered with the International Standard Randomised Controlled Trial registry, ISRCTN84086586, and follow-up is complete.

**Findings:**

From Sept 17, 2014, to May 15, 2017, 3983 eligible children were randomly assigned to treatment, and followed up for 180 days. 164 (4%) were lost to follow-up. 1901 (95%) of 1997 assigned multivitamin multimineral supplement, 1911 (96%) of 1986 assigned iron and folate, and 1922 (96%) of 1994 assigned co-trimoxazole started treatment. By day 180, 166 (8%) children in the multivitamin multimineral supplement group versus 169 (9%) children in the iron and folate group had died (hazard ratio [HR] 0·97, 95% CI 0·79–1·21; p=0·81) and 172 (9%) who received co-trimoxazole versus 163 (8%) who did not receive co-trimoxazole had died (HR 1·07, 95% CI 0·86–1·32; p=0·56). We found no evidence of interactions between these randomisations or with transfusion randomisations (p>0·2). By day 180, 489 (24%) children in the multivitamin multimineral supplement group versus 509 (26%) children in the iron and folate group (HR 0·95, 95% CI 0·84–1·07; p=0·40), and 500 (25%) children in the co-trimoxazole group versus 498 (25%) children in the no co-trimoxazole group (1·01, 0·89–1·15; p=0·85) had had one or more serious adverse events. Most serious adverse events were re-admissions, occurring in 692 (17%) children (175 [4%] with at least two re-admissions).

**Interpretation:**

Neither enhanced supplementation with multivitamin multimineral supplement versus iron and folate treatment or co-trimoxazole prophylaxis improved 6-month survival. High rates of hospital re-admission suggest that novel interventions are urgently required for severe anaemia, given the burden it places on overstretched health services in Africa.

**Funding:**

Medical Research Council and Department for International Development.

## Introduction

Severe anaemia, defined as haemoglobin of less than 6 g/dL, is a leading cause of hospital admission and mortality in children in sub-Saharan Africa.^1^, ^2^, ^3^, ^4^ Outcomes remain unsatisfactory, with high rates of reported in-hospital mortality (9–10%);^2^, ^3^ however, long-term outcomes are also poor, with high additional mortality (8%), anaemia relapse (6%), and re-admission (17%) by 6 months after discharge.^4^ Thus, transfusion alone might not be sufficient to achieve optimal outcomes for these children.

Anaemia is often multifactorial, with several cofactors related to mortality risk. In a comprehensive case-control study of children admitted to hospital with severe anaemia in Africa,^3^ bacteraemia, malaria, hookworm, HIV, or vitamins A and B12 deficiency, or all six, were key associations. Both iron and folate deficiencies were less prevalent among cases than controls, and neither were associated with mortality.^4^ Because WHO already recommend iron, folate, and anthelmintics after discharge for children admitted with severe anaemia,^5^ the clearest potentially modifiable underlying causes of late anaemia recurrence are additional nutritional factors and recurrent bacterial infections. Multivitamin multimineral supplement Nutromix, also known as sprinkles, are widely available globally and offer a low-risk strategy for correcting underlying nutritional anaemia.^6^, ^7^ Similarly, co-trimoxazole prophylaxis has shown mortality benefits (with extremely low rates of toxicity) in children infected with HIV,^8^, ^9^ generally attributed to reductions in bacterial infections, even in areas of high background resistance.^10^, ^11^ Co-trimoxazole might therefore reduce the occurrence of bacterial infections after severe anaemia. However, whether this would affect re-admission or mortality is unclear, particularly because it did not improve outcomes after hospital admission for severe malnutrition.^12^

**Research in context**

**Evidence before this study**

Currently there are no specific recommendations for infection prophylaxis in African children admitted to hospital with severe anaemia that address the high mortality and re-admission rate in the high-risk period after discharge period (3–6 months). A trial in Malawi considered 90 days of malaria chemoprevention consisting of three treatment courses of artemether-lumefantrine (in hospital, and 1 month and 2 months after discharge) for children younger than age 5 years. In the 180 days after discharge, deaths or re-admissions were reduced by 31%. In two trials in The Gambia, malaria chemoprevention during the malaria transmission season in children admitted to hospital with severe anaemia reduced clinical malaria by half and all-cause hospital re-admission by 78%. No trials have investigated antibacterial chemoprophylaxis for African children admitted to hospital with severe anaemia.

The use of iron supplements in Africa is an area of controversy, with a number of observational studies and community-based clinical trials suggesting that iron might increase the risk of malaria. In 2013, a controlled trial showed no increased risk of malaria or hospitalisation for iron-treated children in malaria-endemic Africa. Children included in this trial were enrolled in the community, tested and treated for malaria, and received insecticide-treated bednets. Following a Cochrane review in 2016, WHO now recommends iron can be administered without screening for anaemia or for iron deficiency (if resources do not permit), as long as malaria prevention or efficient management services, or both, are available.

No trial has considered the optimal iron treatment or supplemental strategy for children following admission to hospital with severe anaemia (who might have received an iron-rich transfusion) even though WHO recommends all children should receive a 90-day course of iron and folate (alongside anthelminth treatment).

**Added value of this study**

Our findings highlight the burden that severe anaemia places on health services in Africa, given the substantial proportion of children with severe anaemia that were re-admitted (~17%) to hospital or died (~9%) within the next 6 months, with infectious causes being the main cause of admission. Neither the use of multivitamin multimineral supplement or co-trimoxazole improved poor outcomes in children after discharge from hospital. Because we were able to examine the dose of iron, and found the haemoglobin recovery was similar at all doses of iron and even in children who did not receive a transfusion, our trial also raises questions about whether iron supplementation improves outcomes. We also found no evidence of increased risk of adverse outcomes in those receiving iron without concomitant antimalarial prophylaxis.

**Implications of all the available evidence**

This is the first time that co-trimoxazole, as infection prophylaxis, and MVMM, as a nutritional supplement, have been tested in a clinical trial in children hospitalised with severe anaemia. Our findings suggest that neither multivitamin multimineral supplements nor co-trimoxazole should be provided to children after discharge from hospital after severe anaemia. Our findings also raise questions about whether iron supplementation is beneficial at all in this population. They also challenge the current recommendation advising to give iron therapy or supplementation to children in malarious areas only if children are receiving malaria prophylaxis. The longer-term morbidity burden experienced by children with severe anaemia suggests that future trials should focus on strategies to prevent these adverse outcomes, particularly re-admissions.

We assessed whether multivitamin multimineral supplement or co-trimoxazole prophylaxis given for 3 months after hospital discharge in children admitted for severe anaemia (<6 g/dL) could prevent long-term mortality and re-admission in African children. The results of the transfusion randomisations are reported separately.^13^, ^14^

## Methods

### Study design and participants

From Sept 17, 2014, to May 15, 2017, we enrolled children into a two-stratum, open-label, multicentre, factorial randomised trial in Uganda (three hospitals) and Malawi (one hospital). Eligible children were aged 2 months to 12 years, presenting to hospital with severe anaemia, defined as haemoglobin of less than 6 g/dL. Children with known chronic diseases (renal or liver failure, malignancies, heart failure, or congenital heart disease) or with burns or trauma, and infants who were exclusively breast fed were excluded.^15^ In stratum A, children with severe and complicated anaemia (haemoglobin <4 g/dL [profound anaemia]) or haemoglobin of less than 6 g/dL plus one or more signs of severity or complications (reduced consciousness [ie, coma or prostration], respiratory distress, acute haemoglobinuria or known sickle cell disease) were randomly assigned (1:1) to transfusion with 30 mL/kg versus 20 mL/kg whole blood (15 *vs* 10 mL/kg settled or packed cells, denoted whole blood equivalent). In stratum B, children with uncomplicated severe anaemia (haemoglobin 4–6 g/dL without any severity signs/complications) were randomly assigned 1:1:2 to immediate whole blood equivalent 30 mL/kg versus 20 mL/kg versus no immediate transfusion (control:standard of care) unless or until severity criteria were met).^15^ We have previously described the blood packs used in the trial.^16^ At the same time, children in both strata were randomly assigned using a factorial design to receive 3 months of co-trimoxazole prophylaxis or not, and to 3 months of multivitamin multimineral supplement supplementation (containing 10 mg iron as microencapsulated ferrous folate and 150 μg folic acid daily for all ages) versus 3 months of standard treatment with iron (either 25 mg iron for children <2 years or 60 mg iron for children ≥2 years) and folate (100–400 μg, depending on the local formulation used as standard-of-care at sites). The multivitamin multimineral supplement also contained vitamins A, B1 (thiamine), B2 (riboflavin), B3 (niacin), B6, B12, C, D, zinc, copper, selenium, and iodine, in addition to folic acid and iron (doses, recommended nutrient intake [RNI] for iron,^17^ and is included in the appendix (p 4); provided by Hexagon Nutrition (Exports) Private, Chenai, India). Nutritional treatments or supplements, or both, and co-trimoxazole were prescribed to be given orally once daily, at discharge or from 5 days post admission for children with extended length of stay. Intake was not supervised, but adherence was monitored through pill counts and self-reports of missed pills at follow-up visits. Where previous written consent from parents or legal guardians could not be obtained, ethics committees approved verbal assent with delayed written informed consent as soon as practicable.^18^ The ethics committees of Imperial College London (London, UK), Makerere University (Kampala, Uganda), and the College of Medicine (Blantyre, Malawi) approved the protocol.

### Randomisation and masking

Children with suspected severe anaemia based on presenting symptoms and signs (eg, severe pallor^19^) were screened using a rapid bedside test (HemoCue Hb301 system; HemoCue AB, Angelholm, Sweden^20^) to determine haemoglobin concentration, and clinically assessed for severity, including history of passing dark or red urine (haemoglobinuria)^21^ or known sickle cell disease. Randomisation was stratified by clinical centre and complicated versus uncomplicated severe anaemia. The trial statistician at the Medical Research Council (MRC) Clinical Trials Unit (London, UK) generated and kept the computer-generated sequential randomisation list, generated using variably sized permuted blocks. Trial numbers and randomised allocations were kept inside opaque sealed envelopes (packed at Kilifi, Kenya), together with separate consecutively numbered packs containing case record forms. The link between pack number and trial number was randomised within blocks. At enrolment, trial staff opened the next consecutively numbered pack, which directed them to a sealed randomisation envelope that was within the next 16 envelopes, but was not the next one, ensuring allocation concealment.

An open-label design was used for multivitamin multimineral supplement because the comparator was standard-of-care iron and folate prescribed from local stocks and it was not possible to create an equivalent iron and folate formulation to the active multivitamin multimineral supplement. There was no placebo multivitamin multimineral supplement formulation available, so a double-dummy approach was not feasible. There was also no placebo readily available for the co-trimoxazole formulation donated to the trial. Because children were already being randomly assigned to multivitamin multimineral supplement versus iron and folate and to co-trimoxazole or not, and as the primary endpoint was objective (mortality) and many secondary endpoints were also objective (laboratory values) or severe (re-admission, serious adverse events), masking was not considered essential. The open-label design also allowed the trial to assess real-world adherence. Nurses and doctors were unmasked, but the laboratory tests were done blind to randomisation groups.

### Procedures

Children were managed on general paediatric wards where ventilation facilities were unavailable. Training and basic infrastructural support for emergency care, bedside capillary haemoglobin (HemoCue Hb301 which was quality-controlled daily), glucose and lactate point-of-care tests, and patient monitors were provided. Blood transfusions were free of charge, from the local blood transfusion services, prescreened for standard transfusion transmissible infections^16^ (but not for malaria) and prepared using standard procedures, but did not include leucocyte reduction.^22^ Other than the transfusion randomisation, all children received standard treatments following national guidelines,^23^, ^24^ including anthelmintics.

A structured case report form was completed at admission and at reviews every 30 min during transfusion, then regularly (at 2 h, 4 h, 8 h, 16 h, 24 h, and daily thereafter) during admission. Haemoglobin was assessed using Hemocue every 8 h in the first 24 h, and daily thereafter. After discharge, children were routinely followed up on days 28, 90, and 180 after randomisation for review of clinical status and haemoglobin measurements. Children exited the trial at 180 days. Serious adverse events were actively solicited at every assessment.

### Outcomes

The primary outcome was mortality through 180 days from randomisation, censoring at the minimum of last ascertainment of vital status (intended to be at the 180-day visit) and 183 days (6 months). Secondary outcomes were mortality at 28 days and 90 days, development of severe anaemia after discharge, re-admission to hospital, proportion achieving correction of anaemia (defined by WHO as >9 g/dL), changes in weight and MUAC, at 90 days and 180 days (nutrition), incidence of bacterial infections and malaria overall and at days 28, 90, and 180, serious adverse events, grade 3–4 toxicity of co-trimoxazole, multivitamin multimineral supplement or standard iron and folate,^15^ and costs and cost-effectiveness. Adverse events were graded using the Common Toxicity Criteria for Adverse Events, version 4.0.^25^ An independent endpoint review committee reviewed all deaths masked to randomised group.

### Statistical analysis

3954 children provided more than 80% power to detect a 5% absolute reduction in 180-day mortality in children randomly assigned to enhanced supplementation (multivitamin multimineral supplement or co-trimoxazole) versus standard treatment (iron and folate or no co-trimoxazole; from 25% to 20%), assuming 6% loss to follow-up at 180 days, with two-sided α of 0·013 to allow for four comparisons (appendix p 5). Interim data were reviewed by an independent data monitoring committee (three annual meetings) using Haybittle-Peto criterion (p<0·001). Randomised groups were compared following intention to treat, using log-rank tests or competing-risks methods for time-to-event outcomes, exact tests for binary outcomes, and generalised estimating equations, with independent working correlation for global tests of repeated measures (normal distribution for continuous outcomes, Poisson distribution for binary outcomes to estimate rate ratios). Primary analyses were stratified by randomisation stratification factors. No adjustment was made for multiple testing (appendix pp 5–6). Analyses were done using Stata, version 15.1. This trial is registered with the International Standard Randomised Controlled Trial registry, ISRCTN84086586.

### Role of the funding source

The funder of the study had no role in trial design, data collection, data analysis, data interpretation, or writing of the report. The corresponding author had the final responsibility to submit for publication. ECG and ASW had access to the raw data.

## Results

**Figure 1:**
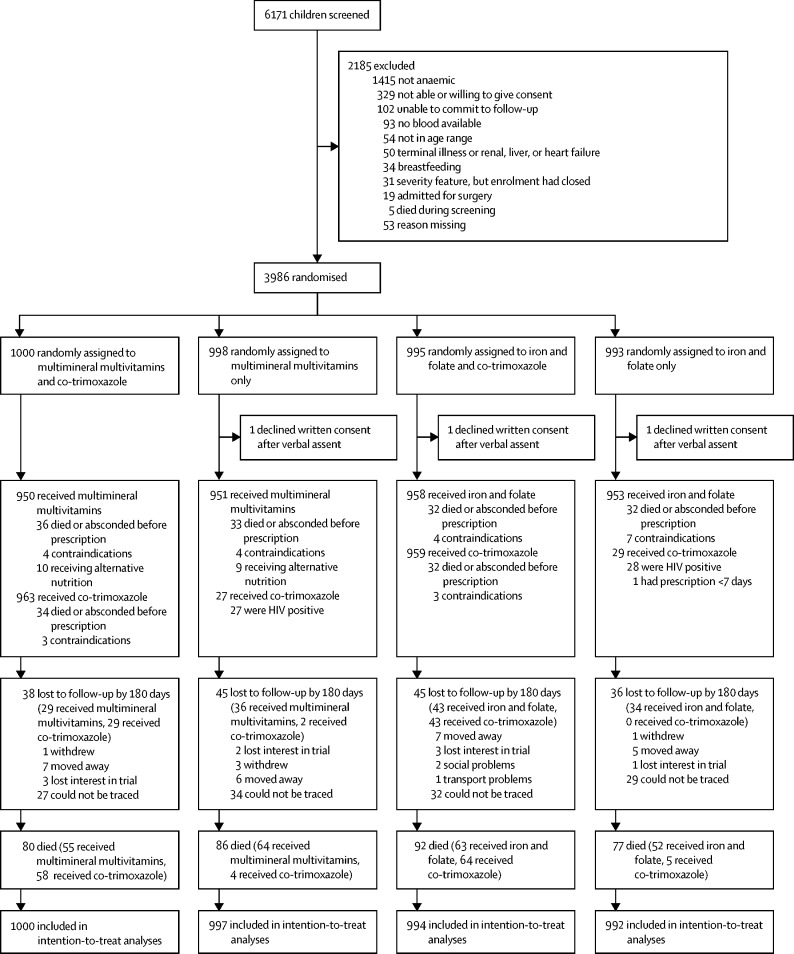
Trial profile All children for whom co-trimoxazole prophylaxis should have been prescribed according to WHO or national guidelines (eg, HIV-infected children) received it regardless of randomisation.

Between Sept 17, 2014, and May 15, 2017, 3986 children with severe anaemia at admission to hospital were randomly assigned to receive enhanced supplementation versus standard treatment; three declined full consent (ie, written consent after verbal assent), and are excluded from all analyses (figure 1). 2418 (61%) of the 3983 included children were from stratum A and had a haemoglobin concentration of less than 4 g/dL or other severity signs or both. Baseline characteristics were balanced between randomised groups (table 1, appendix p 22). Median age was 35 months (IQR 17–61) and haemoglobin was 4·5 g/dL (IQR 3·6–5·3). In the 3983 children enrolled, a history of fever was common (3863, 97%), but fewer children had a documented temperature of more than 37·5°C at screening (1522, 38%) or signs of shock (1170, 29%). 2537 (64%) of 3983 had *Plasmodium falciparum* malaria, but HIV infection (116 [3%] of 3983), microbiologically confirmed bacteraemia (125 [4%] of 3447), and severe malnutrition (75 [2%] of 3983) were uncommon (table 1). After batch genotyping, numbers with sickle cell disease increased from 466 (12%) to 1054 (27%), while 34 (1%) children reported as known sickle cell disease were AA or AS on genotyping.

**Table 1:** Baseline characteristics

		**MVMM (N=1997)**	**Iron and folate (N=1986)**	**Co-trimoxazole (N=1994)**	**No co-trimoxazole (N=1989)**	**Total (N=3983)**
Age, months		36 (17–61)	34 (16–62)	36 (18–63)	34 (17–61)	35 (17–61)
Sex						
	Female	848 (42%)	880 (44%)	855 (43%)	873 (44%)	1728 (43%)
	Male	1149 (58%)	1106 (56%)	1139 (57%)	1116 (56%)	2255 (57%)
Haemoglobin, g/dL		4·5 (3·6–5·4)	4·5 (3·6–5·3)	4·5 (3·6–5·3)	4·5 (3·6–5·3)	4·5 (3·6–5·3)
Weight, kg		11·9 (9·0–15·6)	11·7 (8·9–15·5)	12·0 (9·0–16·0)	11·5 (8·9–15·3)	11·8 (9·0–15·6)
MUAC, cm		14·5 (13·5–15·5)	14·5 (13·5–15·5)	14·5 (13·5–15·5)	14·5 (13·5–15·5)	14·5 (13·5–15·5)
	Severe malnutrition*	31 (2%)	44 (2%)	28 (1%)	47 (2%)	75 (2%)
	Undernourished†	84 (4%)	97 (5%)	95 (5%)	86 (4%)	181 (5%)
Heart rate, beats per minute		146 (130–160)	146 (131–160)	146 (130–160)	146 (131–160)	146 (131–160)
History of fever in this illness		1937 (97%)	1926 (97%)	1937 (97%)	1926 (97%)	3863 (97%)
Axillary temperature‡ at screening		37·2 (36·7–37·9)	37·3 (36·7–38·0)	37·3 (36·7– 38)	37·3 (36·7– 37·9)	37·3 (36·7–38·0)
	Fever, >37·5°C	729 (37%)	793 (40%)	776 (39%)	746 (38%)	1522 (38%)
	Hypothermia, <36·0°C	72 (4%)	75 (4%)	72 (4%)	75 (4%)	147 (4%)
Oxygen saturation		97% (95–99)	98% (95–99)	98% (95–99)	97% (95–99)	97% (95–99)
Respiratory rate, breaths per minute		40 (33–50)	41 (34–50)	40 (33–50)	41 (34–50)	40 (33–50)
Shock§		572 (29%)	598 (30%)	569 (29%)	601 (30%)	1170 (29%)
Severe dehydration (skin turgor or sunken eyes)		135 (7%)	134 (7%)	123 (6%)	146 (7%)	269 (7%)
HIV positive		58/1901 (3%)	57/1884 (3%)	57/1901 (3%)	58/1884 (3%)	115/3785 (3%)
Malaria slide or RDT positive		1262 (63%)	1275 (64%)	1288 (65%)	1249 (63%)	2537 (64%)
Positive blood culture		59/1726 (3%)	66/1721 (4%)	54/1719 (3%)	71/1728 (4%)	125/3447 (4%)
C-reactive protein (mg/dL)		59 (21·1–111·7)	62·3 (24·4–112·9)	61·2 (21·1–111·7)	59·8 (24–113·8)	60·4 (22·7–112·3)
Any severity feature		1217 (61%)	1204 (61%)	1210 (61%)	1211 (61%)	2421 (61%)
Reported sickle cell disease (at screening)		259 (13%)	207 (10%)	232 (12%)	234 (12%)	466 (12%)
Sickle cell disease genotyping¶		542/1980 (27%)	512/1964 (26%)	538/1973 (27%)	518/1971 (26%)	1054/3944 (27%)
	Reported sickle cell; positive genotype	241 (12%)	190 (10%)	217 (11%)	214 (11%)	431 (11%)
	Undiagnosed, sickle cell-positive genotype	301 (15%)	322 (16%)	319 (16%)	304 (15%)	623 (16%)
Country						
	Uganda	1764 (88%)	1765 (88%)	1763 (88%)	1757 (88%)	3520 (88%)
	Malawi	233 (12%)	230 (12%)	231 (12%)	232 (12%)	463 (12%)

Data are n (%), median (IQR), or n/N (%). MMVM=multivitamin multimineral supplements. MUAC=mid-upper arm circumference. RDT=rapid diagnostic test. Weight for height Z score=WHZ. WAZ=weight for age Z score.

* One or more of MUAC <11·0 cm (children aged 2–6 months) or MUAC <11·5 cm (children aged 6–59 months) or WHZ less than −3 (or WAZ if height not recorded) or presence of kwashiorkor at any age.

† One or more of MUAC ≥11·0–11·9 cm (children aged 2–6 months) or MUAC ≥11·5–12·4 cm (children aged 6–59 months) or WHZ −3 to −2 (or WAZ if height not recorded) at any age.

‡ Measured using a digital thermometer.

§ Any one of capillary refill time >2 s, temperature gradient or weak pulse.

¶ From batch genotyping at Kilifi (on samples taken at baseline) after the end of the trial. 39 children had missing results.

1901 (95%) of 1997 children randomly assigned to receive multivitamin multimineral supplement versus 1911 (96%) of 1986 children randomly assigned to receive iron and folate initiated their supplementation and treatment, respectively (appendix pp 23–24), both a median of 4 days (IQR 3–5) after admission. Numbers still reporting taking allocated supplementation and treatment dropped to 1812 (91%) versus 1817 (91%), respectively, at 28 days, and to 1726 (86%) versus 1713 (86%), respectively, at 90 days. At 28 days, significantly more carers reported that children had missed one or more doses of multivitamin multimineral supplement (625, 34%) than iron and folate (460, 25%; p<0·0001), with similar results at 90 days (687 [39%] versus 517 [30%] reporting missing one or more doses since last visit, respectively; p<0·0001). However, most returned relatively few sachets and tablets (appendix p 23–24).

1922 (96%) of 1994 children randomly assigned to co-trimoxazole initiated treatment a median of 4 days (IQR 3–5) days after admission. Numbers still reporting taking co-trimoxazole dropped to 1798 (90%) at 28 days, and to 1690 (85%) at 90 days. At 28 days, 466 (26%) carers reported that children had missed one or more doses of co-trimoxazole, with similar results at 90 days (522, 31%). However, most returned relatively few tablets (appendix p 24–25). Percentages missing doses of co-trimoxazole were similar to percentages missing doses of iron and folate (appendix p 23–24).

**Figure 2:**
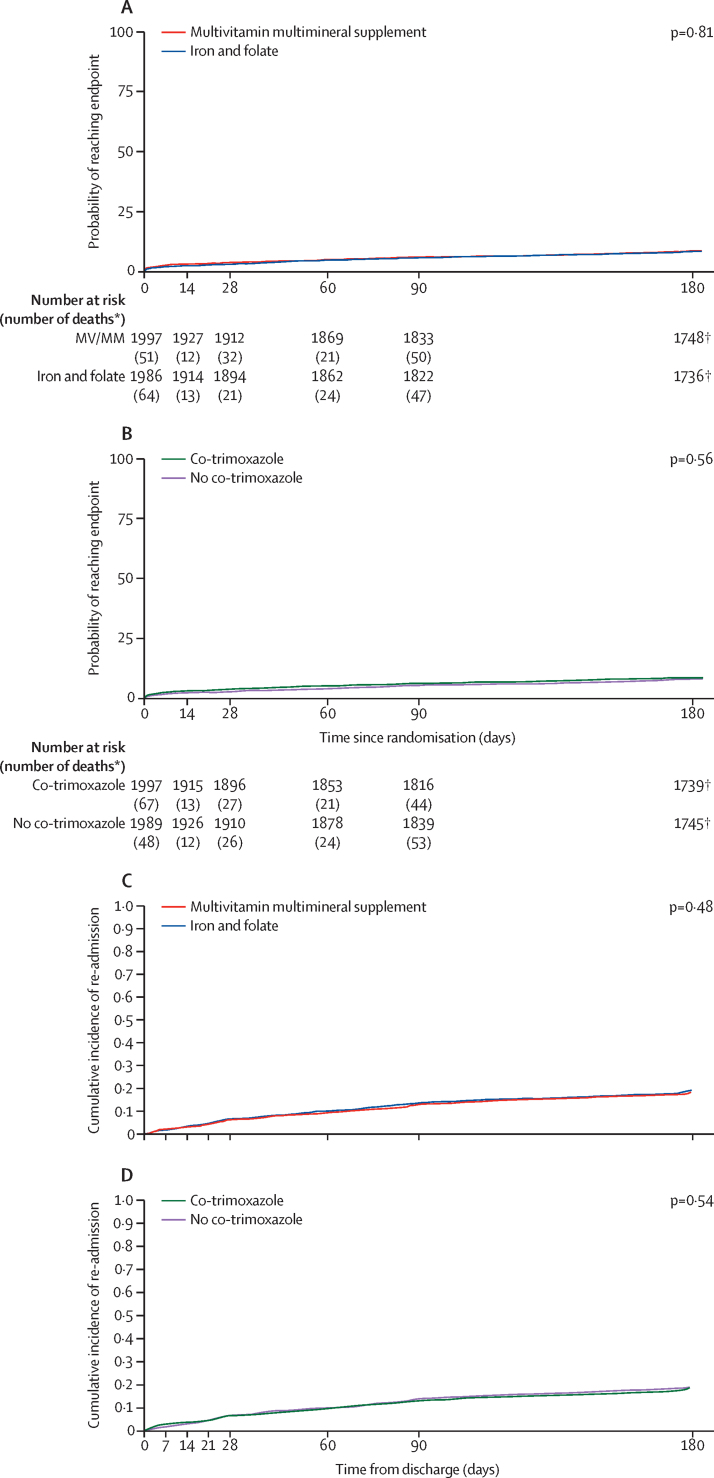
Mortality and re-admissions through 180 days Mortality (A) and re-admission (C) in the multivitamin multimineral supplement and iron and folate groups, and mortality (B) and re-admission (D) in the co-trimoxazole and no co-trimazole groups. *These deaths occurred between timepoints on the x-axis. † Considered to have attended the 180-day visit as seen within the 120–240-day visit window: 3459 (99%) of 3484 seen at day 170 or later.

Vital status at day 180, the primary endpoint, was unknown for 83 (4%) of 1997 children assigned to multivitamin multimineral supplement versus 81 (4%) of 1986 children assigned to iron and folate, and 83 (4%) of 1994 children assigned to co-trimoxazole versus 81 (4%) of 1989 children assigned to no co-trimoxazole. By day 180, 166 (8%) children in the multivitamin multimineral supplement group versus 169 (9%) children in the iron and folate group had died (hazard ratio [HR] 0·97, 95% CI 0·79–1·21; p log-rank=0·81; figure 2; table 2). There was no evidence of interaction with other factorial randomisations (p>0·2). By day 180, 172 (9%) children in the co-trimoxazole group versus 163 (8%) children in the no co-trimoxazole group had died (1·07, 0·86–1·32; p log-rank=0·56; figure 2; table 3), with no evidence of interaction with other factorial randomisations (p>0·2). 169 (48%) of 355 deaths could not be assigned a cause, primarily because they occurred out of hospital with little available information (appendix p 26). The most common assigned primary cause of death was specific infections (58 [16%] of 355), with 53 (15%) attributed to haematological conditions and 23 (6%) to lower respiratory tract infections.

**Table 2:** Secondary and other outcomes in the MVMM randomisation

		**MVMM (N=1997)**	**Iron and folate (N=1986)**	**Total participants (N=3983)**	**Hazard ratio (95% CI)**	**p value**
Death						
	Before prescription	42 (2%)	52 (3%)	94 (2%)	NA	NA
	28 days*	63 (3%)	77 (4%)	140 (4%)	0·81 (0·58–1·13)	0·22
	90 days*	116 (6%)	122 (6%)	238 (6%)	0·94 (0·73–1·22)	0·66
	180 days (primary outcome)	166 (8%)	169 (9%)	335 (8%)	0·97 (0·79–1·21)	0·81
Development of severe anaemia (haemoglobin <6 g/dL) post discharge*		392 (20%)	391 (19%)	783 (20%)	0·99 (0·86–1·13)†	0·88
Readmission to hospital*		339 (17%)	353 (17%)	692 (17%)	0·95 (0·82–1·10)†	0·48
Any serious adverse event*		489 (24%), 670	509 (26%), 681	998 (25%), 1351	0·95 (0·84–1·07)	0·40
	Serious adverse event including anaemia	261 (13%), 352	276 (14%), 370	537 (13%), 722	NA	0·46‡
	Serious adverse event including malaria	146 (7%), 165	142 (7%), 159	288 (7%), 324	NA	0·86‡
	Serious adverse event including sepsis	81 (4%), 103	92 (5%), 110	173 (4%), 213	NA	0·39‡
	Serious adverse event including haemoglobinuria	54 (3%); 66	57 (3%), 65	111 (3%), 131	NA	0·77‡

Data are n (%) or n (%), events, unless otherwise stated. All interaction p values between the factorial randomisations across time-to-event secondary outcomes are p>0·08 (appendix p 33). MVMM=multivitamin multimineral supplements. NA=not applicable.

* Prespecified secondary outcome.

† From competing risks subhazard regression.

‡ Fisher's Exact test.

**Table 3:** Secondary and other outcomes in the co-trimoxazole randomisation

		**Co-trimoxazole (N=1994)**	**No co-trimoxazole (N=1989)**	**Total participants (N=3983)**	**Hazard ratio (95% CI)**	**p value**
Death						
	Before prescription	44 (2%)	NA	NA	NA	NA
	28 days*	80 (4%)	60 (3%)	140 (4%)	1·34 (0·96–1·87)	0·09
	90 days*	128 (6%)	110 (6%)	238 (6%)	1·17 (0·91–1·51)	0·22
	180 days, primary outcome	172 (9%)	163 (8%)	335 (8%)	1·07 (0·86–1·32)	0·56
Development of severe anaemia (haemoglobin <6 g/dL) post discharge*		406 (20%)	277 (19%)	783 (20%)	1·09 (0·95–1·25)†	0·24
Readmission to hospital*		338 (17%)	354 (18%)	692 (17%)	0·95 (0·82–1·11)†	0·54
Any serious adverse event†		500 (25%), 673	498 (25%), 678	998 (25%), 1351	1·01 (0·89–1·15)	0·85
	Serious adverse event including anaemia	280 (14%), 371	257 (13%), 351	537 (13%), 722	NA	0·31‡
	Serious adverse event including malaria	126 (6%), 140	162 (8%), 184	288 (7%), 324	NA	0·03‡
	Serious adverse event including sepsis	99 (5%), 114	74 (4%), 99	173 (4%), 213	NA	0·06‡
	Serious adverse event including haemoglobinuria	50 (3%), 58	61 (3%), 73	111 (3%), 131	NA	0·29‡

Data are n (%) or n (%), events, unless otherwise stated. All interaction p values between the factorial randomisations across time-to-event secondary outcomes are p>0·08 (appendix p 33). NA=not applicable.

* Prespecified secondary outcome.

† From competing risks subhazard regression.

‡ Fisher's Exact test.

For both randomisations, of 11 subgroups prespecified in the protocol (appendix pp 8, 10), and five prespecified in the statistical analysis plan (appendix pp 9, 11), only one (malnutrition) showed evidence of heterogeneity in the effect of multivitamin multimineral supplement versus iron and folate (p=0·01; rest p>0·1). However, this was driven by undernourished and malnourished subgroups, which were very small, and might be expected by chance because of the number of subgroups tested (n=31 across both randomisations). Malnutrition was defined using MUAC; considering MUAC as a continuous measure showed no evidence of heterogeneity in the effect of multivitamin multimineral supplement versus iron and folate (p=0·57).

Children were discharged a median of 4 days (IQR 3–5) after admission in all randomised groups. Day 180 visits were completed for 1730 (87%) of 1997 children in the multivitamin multimineral supplement group versus 1720 (87%) of 1986 children in the iron and folate group, and 1719 (86%) of 1994 children in the co-trimoxazole group versus 1731 (87%) of 1989 children in the no co-trimoxazole group.

339 (17%) of 1997 children in the multivitamin multimineral supplement group versus 353 (17%) of 1986 children in the iron and folate group were re-admitted within 180 days of randomisation (HR 0·96, 95% CI 0·83–1·11; p=0·59; figure 2; table 2). 89 (4%) versus 86 (4%), respectively, were re-admitted two or more times before 180 days. Children increased their weight and MUAC post discharge, but there was no evidence that changes differed between multivitamin multimineral supplement and iron and folate groups at 90 days (p=0·22 and p=0·48, respectively) or 180 days (p=0·65 and p=0·36, respectively; table 4).

**Table 4:** Changes in weight and MUAC in the MVMM randomisation

	**MVMM**	**Iron and folate**	Difference*
**90 days**			
Change in weight from baseline, kg	1·25 (1·19 to 1·31), N=1764	1·19 (1·13 to 1·25), N=1758	0·05 (−0·03 to 0·14), p=0·22
Change in MUAC from baseline, cm	0·47 (0·42 to 0·51), N=1770	0·46 (0·42 to 0·51), N=1760	0·02 (−0·04 to 0·08), p=0·48
**180 days**			
Change in weight from baseline, kg	1·82 (1·75 to 1·88), N=1676	1·79 (1·72 to 1·86), N=1682	0·02 (−0·07 to 0·12), p=0·65
Change in MUAC from baseline, cm	0·62 (0·58 to 0·66), N=1696	0·66 (0·62 to 0·71), N=1693	−0·03 (−0·09 to 0·03), p=0·36

Data are mean (95% CI), N or mean (95% CI), p value. MVMM=multivitamin multimineral supplements. MUAC=mid-upper arm circumference.

* Estimated differences and confidence intervals obtained from a linear regression adjusted for baseline values.

338 (17%) of 1994 children in the co-trimoxazole group versus 354 (18%) of 1989 children in the no co-trimoxazole group were re-admitted within 180 days of randomisation (HR 0·96, 95% CI 0·83–1·11; p=0·61; table 3; figure 2). 84 (4%) versus 91 (5%), respectively, were re-admitted two or more times before 180 days. Children increased their weight and MUAC post discharge, but there was no evidence that changes differed between co-trimoxazole and no co-trimoxazole groups at 90 days (p=0·13 and p=0·92, respectively) or 180 days (p=0·25 and p=0·40 respectively; table 5).

**Table 5:** Changes in weight and MUAC in the co-trimoxazole randomisation

	**Co-trimoxazole**	**No co-trimoxazole**	Difference*
**90 days**			
Change in weight from baseline, kg	1·26 (1·20 to 1·32), N=1754	1·19 (1·13 to 1·25), N=1768	0·07 (−0·02 to 0·15), p=0·13
Change in MUAC from baseline, cm	0·45 (0·41 to 0·50), N=1754	0·48 (0·43 to 0·52), N=1776	0·00 (−0·05 to 0·06), p=0·92
**180 days**			
Change in weight from baseline, kg	1·84 (1·77 to 1·91), N=1669	1·78 (1·71 to 1·84); N=1689	0·06 (−0·04 to 0·15); p=0·25
Change in MUAC from baseline, cm	0·62 (0·57 to 0·67); N=1685	0·66 (0·62 to 0·71); N=1704	−0·03 (−0·09 to 0·04); p=0·40

Data are mean (95% CI), N or mean (95% CI), p value. MUAC=mid-upper arm circumference.

* Estimated differences and confidence intervals obtained from a linear regression adjusted for baseline values.

489 (24%) of 1997 children in the multivitamin multimineral supplement group versus 509 (26%) of 1986 children in the iron and folate group had one or more serious adverse events (p=0·40; table 2; appendix p 27–32). In the multivitamin multimineral and iron and folate groups, respectively, 670 versus 681 serious adverse events occurred, of which 475 versus 491 were re-admissions to hospital. There was no evidence of differences between the two groups in anaemia, malaria, sepsis, or haemoglobinuria serious adverse events (p>0·3; table 2). 500 (25%) of 1994 children in the co-trimoxazole group versus 498 (25%) of 1989 children in the no co-trimoxazole group had one or more serious adverse event (p=0·85; table 3; appendix p 27–32), for a total number of events of 673 versus 678, of which 472 versus 494 were re-admissions to hospital, respectively. There was no evidence of differences between co-trimoxazole versus no co-trimoxazole in anaemia or haemoglobinuria serious adverse events (p>0·28) and weak evidence for a difference in sepsis serious adverse events (p=0·06), but children randomly assigned to co-trimoxazole had significantly fewer malaria serious adverse events (p=0·03; table 3). There was no evidence of interactions between randomisations for these other secondary outcomes (p_heterogeneity_>0·08, appendix p 33).

After discharge, there was no evidence of difference between the multivitamin multimineral supplement and iron and folate groups in the proportions of patients with positive malaria slides (p>0·4; appendix p 12), with possible malaria infection (positive malaria slide or reporting having had an acute febrile illness or having taken antimalarials; p>0·3; appendix p 13) or possible bacterial infection (severe infection, or reporting having received antibiotics or having an acute febrile illness; p>0·4; appendix p 14) or severe bacterial infection (p=0·82; appendix p 15). At 28 days, co-trimoxazole reduced the proportions of patients with positive malaria slides (p<0·001; appendix p 12), and possible malaria infection (p=0·02; appendix p 13), but there was no evidence of differences at days 90 or 180 (p>0·1), and no evidence of differences in possible or severe bacterial infections (p>0·1; appendix pp 14–15).

Few grade 3–4 adverse events for co-trimoxazole, multivitamin multimineral supplement, or standard iron and folate were reported as probably or possibly related by site clinicians (co-trimoxazole n=10; multivitamin multimineral supplement n=4; iron and folate n=1; appendix p 34). None were judged to be related on independent clinical review. Results of primary and secondary outcomes restricted to patients alive at the minimum of discharge or 5 days from randomisation in whom the interventions were neither mandated nor contraindicated were similar (appendix p 35).

**Figure 3:**
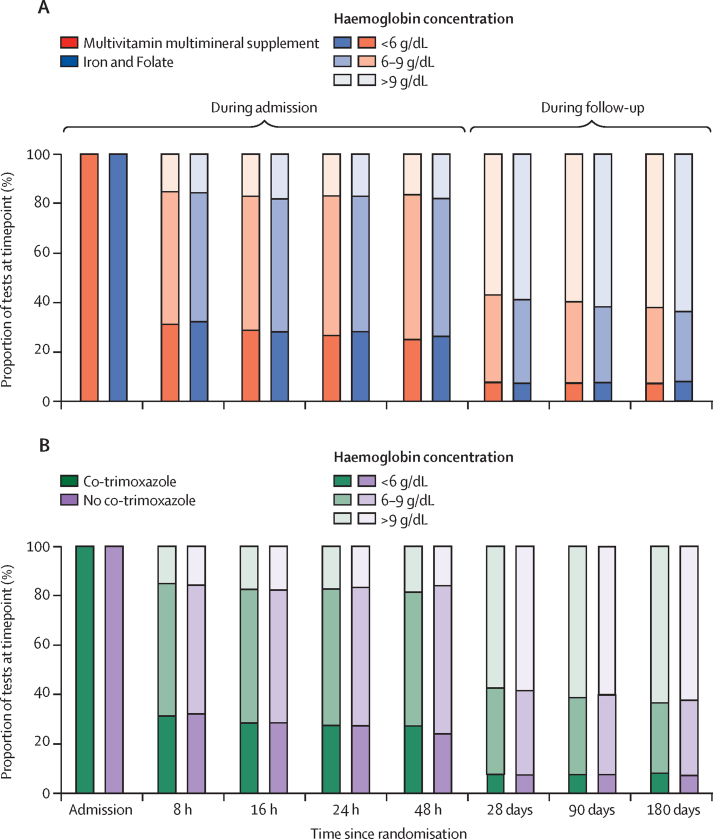
Proportions of children with haemoglobin concentrations of less than 6 g/dL, 6–9 g/dL, and more than 9 g/dL over 180 days in multivitamin multimineral supplement and iron and folate groups (A) and co-trimoxazole and no co-trimazole groups (B) Numbers can be found in the appendix (p 38).

There was no evidence of difference between the multivitamin multimineral supplement versus iron and folate groups in the development of severe anaemia; p=0·16; table 2) or in the proportions achieving correction of anaemia (>9 g/dL) after discharge (p>0·2; figure 3; appendix p 38), or in the mean change in haemoglobin from baseline (p>0·2; appendix p 16). There was also no evidence of difference between co-trimoxazole versus no co-trimoxazole in the development of severe anaemia after discharge (p=0·87, table 3), or in the proportions of children achieving correction of anaemia (>9 g/dL; figure 3) after discharge (p>0·3), or in the mean change in haemoglobin from baseline (p>0·4; appendix p 17). Overall, 2871 (74%) children had already achieved a haemoglobin concentration of at least 6 g/dL by 48 h and 665 (17%) children had achieved a concentration of more than 9 g/dL (figure 3; appendix pp 16–17, 38). Discharge haemoglobin concentrations were not available, but by 28 days after discharge, they had increased to 3352 (92%) and 2100 (58%), respectively, and subsequently remained fairly stable.

To explore the effect of iron dose, we did exploratory subgroup analyses by age group. Children younger than age 2 years (1400, 35%) received 10 mg of iron if randomly assigned to multivitamin multimineral supplement versus 25 mg of iron if randomly assigned to iron and folate; children older than age 2 years received 10 mg versus 60 mg of iron, respectively. Because even 60 mg of iron might be subtherapeutic in children older than 5 years, we considered children aged 2–5 years (2459, 62%) and children older than 5 years (124, 3%) separately. Firstly, in this exploratory analysis there was no evidence of heterogeneity in the lack of effect of multivitamin multimineral supplement versus iron and folate on mortality at day 180 across these age groups (p_heterogeneity_=0·96; HR 0·97, 95% CI 0·67–1·40 for <2 years; HR 0·95, 0·73–1·26 for 2–5 years; and HR 1·09, 0·42–2·81 for >5 years), and no evidence of an interaction for serious adverse events (p_heterogeneity_=0·62). Furthermore, despite the substantial difference in iron doses received in the different age groups, haemoglobin recovery was similar in children receiving multivitamin multimineral supplement and iron and folate in each of the age groups (appendix p 18).

To assess whether this effect could be driven by the fact that many children received a transfusion during the trial, and hence iron absorption from the transfusion might have addressed underlying iron deficiency, in further exploratory analyses we compared haemoglobin recovery in children in the stratum with a haemoglobin concentration of 4–6 g/dL, and no severity signs randomised to immediate versus triggered transfusion, splitting the triggered transfusion group by (non-randomised) receipt of transfusion (appendix p 19). Adjusting for predictors of receiving a transfusion, there was no evidence that the lack of effect of multivitamin multimineral supplement versus iron and folate on haemoglobin recovery at days 28, 90, or 180 varied by whether or not, and when, a transfusion was received (p>0·6). In a final exploratory analysis, we considered the effect of age subgroup (as a proxy for iron dose) on malaria (appendix p 20), but found no evidence that the lack of effect of multivitamin multimineral supplement versus iron and folate varied by age group for either slide positive malaria (p_heterogeneity_>0·20) or possible malaria (p>0·45). In particular, higher rates of malaria with higher iron doses were not evident within any of these subgroups at any timepoint (p>0·2).

## Discussion

In this large multicentre trial, we observed no overall differences in mortality over 180 days among children with severe anaemia receiving multivitamin multimineral supplement versus iron and folate treatment or receiving co-trimoxazole prophylaxis. the only evidence for differences across various outcomes were reductions in malaria and in sepsis serious adverse events in children randomly assigned to co-trimoxazole. Overall, we found that children admitted with severe anaemia were at high risk of poor outcomes, including 8% mortality within 6 months of admission (6% in the first 90 days), and high rates of re-admission post discharge (17%). Establishing that novel interventions are urgently required for this high-risk group of children.

This was a pragmatic trial testing the clinical effectiveness of practical interventions that could be implemented at scale, rather than the efficacy of the drugs. The strength and generalisability of the trial lies in its broad eligibility criteria, high adherence to randomised interventions, and completeness of follow-up to 28 days and 180 days. Large subgroups of children with malaria and sickle cell disease were included, making the absence of benefit generalisable to areas of Africa where these conditions are prevalent. Although outcomes in children with sickle cell disease have recently been recognised as a major burden on health services,^26^ in our study, mortality of children in sickle cell disease was lower than in children without sickle cell disease, suggesting that the disease did not disproportionately affect our results. Limitations of the study include the lack of definitive diagnoses in many children dying at home and that mortality was lower than anticipated, possibly due to the provision of standard medications for all participants by the trial and the close contact with the patients during follow-up so new illnesses requiring re-admission were identified early in their course. Nevertheless, the 95% CI suggested that relative reductions of more than 20% (absolute benefits of >1·8%) associated with the interventions can be reliably excluded given the results of this trial. However, the number of re-admissions were substantial (which might possibly have averted some deaths), and so the trial had good power to detect modest effects on this outcome. Although in the protocol, we were unable to obtain haemoglobin concentration at discharge for most children because many parents refused this test after children recovered. the substantial increases in haemoglobin concentration from 48 h to 28 days post admission (appendix pp 16–17) makes it difficult to assess the optimal time for future interventions to target. We did not have funding to estimate micronutrient deficiencies at baseline. We did not include a placebo control for either intervention; the inclusion of such a control could have affected attribution of illness episodes to infection for co-trimoxazole prophylaxis, although rates were constant over follow-up, before and after co-trimoxazole prophylaxis was being prescribed (appendix p 15). Adherence was imperfect, with around one-third of participants reporting missing any doses, although the numbers of tablets returned suggested that most were only missing occasional doses. This proportion of adherence probably reflects usual practice by carers and children. We found expected effects of co-trimoxazole on malaria, sepsis, and fevers, which suggests that sufficient active ingredient was being ingested to identify an effect. Lastly, given the scarcity of available resources, randomisation was done at admission, at the same time as the transfusion randomisations. The fact that a small proportion of children (3%, appendix p 23) died or absconded before they could start the intervention means that our intention-to-treat estimates are slightly biased towards the null compared with those that would have been obtained from a trial randomising at discharge, but the factorial design ensures balance across the transfusion interventions.

Iron supplementation and treatment has been controversial in children in malaria-endemic areas. A community-based randomised controlled trial in Pemba, Zanzibar, showed a higher risk for serious events including death or admission to hospital with malaria in iron (12·5 mg) plus folic acid versus placebo (actual difference 12%, 95% CI 2–23%),^27^ resulting in recommendations that iron and folic acid should be targeted to children with anaemia who are at risk of iron deficiency.^28^, ^29^ However, the diagnosis of iron deficiency is unreliable in children who are febrile with an acute inflammatory state secondary to infection.^30^, ^31^, ^32^ Furthermore, owing to the significant burden that testing children for iron status places on health-care services, WHO revised their recommendations in 2011, indicating children receiving iron in malaria-endemic areas should be concurrent with “measures to prevent, diagnose and treat malaria”.^33^ This approach was subsequently supported by a trial from Ghana^34^ and reinforced by a Cochrane systematic review^35^ showing no overall harm for children treated with iron if given alongside malaria diagnosis, treatment, and insecticide-treated bednets. In TRACT we compared multivitamin multimineral supplement containing the equivalent of supplementary iron and folate doses to standard treatment doses in four centres with differing levels of malaria endemicity. Half of the children were also randomly assigned to receive concurrent infection prophylaxis with co-trimoxazole. We found no evidence of differences in haemoglobin recovery by nutritional strategy (appendix p 18) nor evidence of an increased risk of mortality or serious adverse events (predominantly hospitalisation or death) or malaria (appendix p 20) in children receiving higher doses of iron, or any interactions in children receiving infection prophylaxis or not. We also found no interaction with transfusion received (including dose) or not (appendix pp 8, 10), and haemoglobin recovery occurred similarly with multivitamin multimineral supplement and iron and folate even in children who were not transfused (appendix p 19).

We found no evidence that supplementation to reduce deficiencies previously identified as associated with severe anaemia^3^ had any effect on any outcome; furthermore, contrary to other reports,^36^ multivitamin multimineral supplement was significantly less acceptable to carers and children than standard iron and folate. Variation in the overall dose of iron received by randomised intervention and by age allowed us to make two important observations. First, in children living in malarious areas, we saw no demonstrable increase in the risks of infection (including malaria and severe sepsis) or death in children receiving higher doses. Second, iron appeared to have a limited role in recovery from severe anaemia. The failure of orally administered iron to improve haematological indices or outcomes in this population might be due to inhibition of ferroportin-mediated systemic iron absorption.^37^ In the presence of infection and inflammation, hepatic hepcidin production is upregulated, effectively causing iron to remain trapped within enterocytes and ultimately be excreted faecally. Although this questions the merit of orally administered iron (or micronutrients) in the management of severe anaemia we did not include a control and placebo group because iron and folate is considered standard treatment.

The choice of co-trimoxazole for infection prophylaxis, rather than co-trimoxazole plus an antimalarial, was based on three considerations. First, as in HIV-infected children,^10^ we hypothesised that it might improve long-term morbidity and mortality by preventing recurrent bacterial infections, a major risk factor in this population. Second, prophylactic co-trimoxazole reduces malaria infection,^38^, ^39^ a key risk factor for recurrence of severe anaemia. Finally, a single dose daily of a medicine is preferable to polypharmacy, where the ideal regimen for both bacterial and malaria prophylaxis in this large heterogenous population is unknown. For example, in children with sickle cell disease (25% of this trial cohort), routine prophylaxis for malaria prevention would use daily sulfadoxine and pyrimethamine; if used with co-trimoxazole, this might lead to overdosing children on sulphonamides, which have long half-lives and are present in both sulphadoxine and pyrimethamine, and co-trimoxazole. However, despite the hypothesised benefits, it is notable that co-trimoxazole prophylaxis appears to be protective only in children with HIV,^8^, ^40^ with no evidence of mortality benefits now having been robustly shown in trials in severe malnutrition^12^ and severe anaemia, despite both showing reductions in some infection events that nevertheless did not translate into mortality reductions. This might be related to deficits in memory CD4 T cells in children infected with HIV even after starting antiretroviral therapy and benefits of co-trimoxazole on inflammation.^41^ Whether an alternative antibiotic such as azithromycin^42^ might have greater effects is unclear,^43^ particularly since the burden of non-malarial febrile illness appeared relatively low.

In conclusion, although our findings highlight the burden that severe anaemia continues to place on overstretched health services in Africa, they do not support the use of multivitamin multimineral supplement, iron and folate, or co-trimoxazole to improve poor outcomes after discharge in children. Because we were able to examine dose of iron and found haemoglobin recovery to be similar at all doses and even in children who did not receive a transfusion, our trial also raises questions about the use of iron treatment at all, calling for future trials incorporating a placebo or control group into the micronutrient arm of a study.

## Data sharing

The TRACT trial data are held at MRC Clinical Trials Unit at University College London (London, UK), which encourages optimal use of data by employing a controlled access approach to data sharing, incorporating a transparent and robust system to review requests, and provide secure data access consistent with the relevant ethics committee approvals. All requests for data are considered and can be initiated by contacting mrcctu.ctuenquiries@ucl.ac.uk.

## References

[1] Stevens GA, Finucane MM, De-Regil LM (2013). Global, regional, and national trends in haemoglobin concentration and prevalence of total and severe anaemia in children and pregnant and non-pregnant women for 1995–2011: a systematic analysis of population-representative data. Lancet Glob Health.

[2] Pedro R, Akech S, Fegan G, Maitland K (2010). Changing trends in blood transfusion in children and neonates admitted in Kilifi District Hospital, Kenya. Malar J.

[3] Calis JC, Phiri KS, Faragher EB (2008). Severe anemia in Malawian children. N Engl J Med.

[4] Phiri KS, Calis JC, Faragher B (2008). Long term outcome of severe anaemia in Malawian children. PLoS One.

[5] WHO (2013). Pocket book of hospital care for children: second edition Guidelines for the management of common childhood illnesses.

[6] Zlotkin S, Antwi KY, Schauer C, Yeung G (2003). Use of microencapsulated iron(II) fumarate sprinkles to prevent recurrence of anaemia in infants and young children at high risk. Bull World Health Organ.

[7] Christofides A, Asante KP, Schauer C, Sharieff W, Owusu-Agyei S, Zlotkin S (2006). Multi-micronutrient Sprinkles including a low dose of iron provided as microencapsulated ferrous fumarate improves haematologic indices in anaemic children: a randomized clinical trial. Matern Child Nutr.

[8] Chintu C, Bhat GJ, Walker AS (2004). Co-trimoxazole as prophylaxis against opportunistic infections in HIV-infected Zambian children (CHAP): a double-blind randomised placebo-controlled trial. Lancet.

[9] Church JA, Fitzgerald F, Walker AS, Gibb DM, Prendergast AJ (2015). The expanding role of co-trimoxazole in developing countries. Lancet Infect Dis.

[10] Mulenga V, Ford D, Walker AS (2007). Effect of cotrimoxazole on causes of death, hospital admissions and antibiotic use in HIV-infected children. AIDS.

[11] Musiime V, Cook A, Bakeera-Kitaka S (2013). Bacteremia, causative agents and antimicrobial susceptibility among HIV-1 infected children on antiretroviral therapy in Uganda and Zimbabwe. Pediatr Infect Dis.

[12] Berkley JA, Ngari M, Thitiri J (2016). Daily co-trimoxazole prophylaxis to prevent mortality in children with complicated severe acute malnutrition: a multicentre, double-blind, randomised placebo-controlled trial. Lancet Glob Health.

[13] Maitland K, Kiguli S, Olupot-Olupot P (2019). Immediate transfusion in African children with uncomplicated severe anemia. New Engl J Med.

[14] Maitland K, Olupot-Olupot P, Kiguli S (2019). Transfusion volume for children in African with severe anemia. N Engl J Med 2019.

[15] Mpoya A, Kiguli S, Olupot-Olupot P (2015). Transfusion and treatment of severe anaemia in African children (TRACT): a study protocol for a randomised controlled trial. Trials.

[16] Uyoga S, Mpoya A, Olupot-Olupot P (2019). Haematological quality and age of donor blood issued for paediatric transfusion to four hospitals in sub-Saharan Africa. Vox Sang.

[17] WHO, Food and Agriculture Organization of the UN (2004). Vitamin and mineral requirements in human nutrition: report of a joint FAO/WHO expert consultation.

[18] Maitland K, Molyneux S, Boga M, Kiguli S, Lang T (2011). Use of deferred consent for severely ill children in a multi-centre phase III trial. Trials.

[19] Olupot-Olupot P, Prevatt N, Engoru C (2019). Evaluation of the diagnostic accuracy and cost of different methods for the assessment of severe anaemia in hospitalised children in Eastern Uganda. Wellcome Open Res.

[20] Medina Lara A, Mundy C, Kandulu J, Chisuwo L, Bates I (2005). Evaluation and costs of different haemoglobin methods for use in district hospitals in Malawi. J Clin Path.

[21] Olupot-Olupot P, Engoru C, Uyoga S (2017). High frequency of blackwater fever among children presenting to hospital with severe febrile illnesses in Eastern Uganda. Clin Infect Dis.

[22] Ala F, Allain JP, Bates I (2012). External financial aid to blood transfusion services in sub-Saharan Africa: a need for reflection. PLoS Med.

[23] Uganda National Clinical Guidelines 2016 (2003). National guidelines for management of common conditions. https://health.go.ug/publications/guidelines.

[24] Phillips JA, Kazembe PN, Nelson EAS, Fisher JAF, Grabosch E (2008). A paediatric handbook for Malawi.

[25] US Department of Health and Human Services (2010). Common Terminology Criteria for Adverse Events (CTCAE) Version 4.03. https://www.eortc.be/services/doc/ctc/CTCAE_4.03_2010-06-14_QuickReference_5x7.pdf.

[26] Macharia AW, Mochamah G, Uyoga S (2018). The clinical epidemiology of sickle cell anemia In Africa. Am J Hematol.

[27] Sazawal S, Black RE, Ramsan M (2006). Effects of routine prophylactic supplementation with iron and folic acid on admission to hospital and mortality in preschool children in a high malaria transmission setting: community-based, randomised, placebo-controlled trial. Lancet.

[28] WHO (2016). Guideline: daily iron supplementation in infants and children. https://www.who.int/nutrition/publications/micronutrients/guidelines/daily_iron_supp_childrens/en/.

[29] Esan MO, van Hensbroek MB, Nkhoma E (2013). Iron supplementation in HIV-infected Malawian children with anemia: a double-blind, randomized, controlled trial. Clin Infect Dis.

[30] Phiri KS, Calis JC, Siyasiya A, Bates I, Brabin B, van Hensbroek MB (2009). New cut-off values for ferritin and soluble transferrin receptor for the assessment of iron deficiency in children in a high infection pressure area. J Clin Pathol.

[31] Nyakeriga AM, Troye-Blomberg M, Dorfman JR (2004). Iron deficiency and malaria among children living on the coast of Kenya. J Infect Dis.

[32] Zimmermann MB, Chassard C, Rohner F (2010). The effects of iron fortification on the gut microbiota in African children: a randomized controlled trial in Cote ‘Ivoire. Am J Clin Nutr.

[33] WHO. Guideline: use of multiple micronutrient powders for home fortification of foods consumed by infants and children 6–23 months of age. Geneva: World Health Organization. 24501787

[34] Zlotkin S, Newton S, Aimone AM (2013). Effect of iron fortification on malaria incidence in infants and young children in Ghana: a randomized trial. JAMA.

[35] Neuberger A, Okebe J, Yahav D, Paul M (2016). Oral iron supplements for children in malaria-endemic areas. Cochrane Database Syst Rev.

[36] Jefferds ME, Ogange L, Owuor M (2010). Formative research exploring acceptability, utilization, and promotion in order to develop a micronutrient powder (Sprinkles) intervention among Luo families in western Kenya. Food Nutr Bull.

[37] Ganz T, Nemeth E (2015). Iron homeostasis in host defence and inflammation. Nat Rev Immunol.

[38] Thera MA, Sehdev PS, Coulibaly D (2005). Impact of trimethoprim-sulfamethoxazole prophylaxis on falciparum malaria infection and disease. J Infect Dis.

[39] Mwenya DM, Charalambous BM, Phillips PP (2010). Impact of cotrimoxazole on carriage and antibiotic resistance of Streptococcus pneumoniae and Haemophilus influenzae in HIV-infected children in Zambia. Antimicrob Agents Chemother.

[40] Bwakura-Dangarembizi M, Kendall L, Bakeera-Kitaka S (2014). A randomized trial of prolonged co-trimoxazole in HIV-infected children in Africa. N Engl J Med.

[41] Bourke CD, Gough EK, Pimundu G, et al. Cotrimoxazole reduces systemic inflammation in HIV infection by blunting immune cell activation and modulating the gut microbiota. *Sci Transl Med* (in press). 10.1126/scitranslmed.aav0537 PMC6783302 30944164

[42] Keenan JD, Bailey RL, West SK (2018). Azithromycin to reduce childhood mortality in sub-Saharan Africa. N Engl J Med.

[43] Pavlinac PB, Singa BO, John-Stewart GC (2017). Azithromycin to prevent post-discharge morbidity and mortality in Kenyan children: a protocol for a randomised, double-blind, placebo-controlled trial (the Toto Bora trial). BMJ Open.

