# Supplementary material for: Co-trimoxazole or multivitamin multimineral supplement for post-discharge outcomes after severe anaemia in African children: a randomised controlled trial
Source: Lancet Glob Health. 2019 Sep 16;7(10):e1435–47. doi: 10.1016/S2214-109X(19)30345-6 (PMC7024999; doi:10.1016/S2214-109X(19)30345-6)
Supplement: Supplementary appendix [file mmc1.pdf]

THE LANCET
Global Health

Supplementary appendix

This appendix formed part of the original submission and has been peer reviewed. We post it as supplied by the authors.

Supplement to: Maitland K, Olupot-Olupot P, Kiguli S, et al. Co-trimoxazole or multivitamin multimineral supplement for post-discharge outcomes after severe anaemia in African children: a randomised controlled trial. *Lancet Glob Health* 2019; **7:** e1435–47.

CONTENTS

SUPPLEMENTARY METHODS 3
(a) TRACT trial group 3
(b) Trial sites 3
(c) Further details of randomisation to cotrimoxazole 3
(d) Further details of randomisation to multi-mineral multivitamins (MVMM) and iron/folate 4
(e) Further details of allocation concealment 4
(f) Further details of endpoint ascertainment and adjudication 5
(g) Sample size calculation from the trial protocol 5
(h) Statistical methods 5
(i) Subgroup analyses 6
Figure S1 Eleven subgroup analyses pre-specified in the protocol for the 180-day mortality primary outcome: MVMM randomisation 8
Figure S2 Five subgroup analyses pre-specified in the Statistical Analysis Plan but not the protocol for the 180-day mortality primary outcome: MVMM randomisation 9
Figure S3 Ten subgroup analyses pre-specified in the protocol for the 180-day mortality primary outcome: cotrimoxazole randomisation 10
Figure S4 Five subgroup analyses pre-specified in the Statistical Analysis Plan but not the protocol for the 180-day mortality primary outcome: cotrimoxazole randomisation 11
Figure S5 Positive malaria slide 12
(a) MVMM 12
(b) Cotrimoxazole 12
Figure S6 Possible malaria infection* 13
(a) MVMM 13
Figure S7 Possible bacterial infection* 14
(a) MVMM 14
(b) Cotrimoxazole 14
Figure S8 Time to new severe bacterial infection 15
(a) MVMM 15
(b) Cotrimoxazole 15
Figure S9 Haemoglobin recovery over 180 days 16
(a) MVMM randomisation 16
(b) Cotrimoxazole randomisation 17
Figure S10 Impact of MVMM on haemoglobin recovery over 180 days by age group (with varying dosage of iron). 18
Figure S11 Impact of MVMM on haemoglobin recovery post-discharge by receipt of blood transfusion in TRACT B only 19
Figure S12 Impact of MVMM on malaria by age group (varying dosage of iron) 20
(a) Positive malaria slide 20
(b) Possible malaria infection* 21
SUPPLEMENTARY TABLES 22
Table S1 Additional baseline characteristics of randomised children 22
Table S2 Prescription and adherence 23

(a) MVMM vs iron/folate 23
(b) cotrimoxazole 24
Table S3 Causes of death as adjudicated by the Endpoint Review Committee 26
Table S4 All SAEs 27
Table S5 Heterogeneity tests between different factorial randomisations for primary and secondary time-to-event outcomes 33
Table S6 Grade 3 or 4 toxicity adverse events that were definitely, probably or possibly related as assigned by the clinician 34
Table S7 Secondary and other clinical outcomes within secondary population 35
Table S8 Changes in weight and MUAC within the secondary population 37
Table S9 Numbers with haemoglobin <6 and >9 g/dl over time from randomisation 38

SUPPLEMENTARY METHODS

(a) TRACT trial group

The TRACT trial group consists of:

Participating Centres:
Department of Paediatrics, Mulago Hospital, Makerere University, Kampala, Uganda: S Kiguli, R O Opoka, E Nabawanuka, J Kayaga, C Williams Musika, E Kadama, I Mbwali, L Nuwabaine, R Nakikwaku, J Nsubuga, K Mpande, R Adoo, O Ouma, N K Adia.

Mbale Regional Referral Hospital Mbale, Uganda: P Olupot-Olupot, J Nteziyaremye, C Namanyanga, G Passi, T, Sennyondo, R Adong, CB Okalebo, E Atimango, S Mwamula, J Kapsindet, G Kiluli R Muhindo, G Masifa N Thembo, G Odong.

Soroti Regional Referral Hospital Mbale, Uganda: C Engoru, F Aloroker, M Nakuya, D Aromut M Ariima, M Itipe, MG Atim, M Abeno, B Amede, M Olupot, S Okwi, MG Kulume, G Among, P Onyas, ED Achipa

KEMRI Wellcome Trust Research Programme, Kilifi, Kenya (coordinating centre for the trial and genetics group): K Maitland, A Mpoya, P Maitha, S Uyoga, TN Williams, A Macharia

College of Medicine and Malawi-Liverpool-Wellcome Trust Clinical Research Programme, College of Medicine, Blantyre, Malawi: M Mallewa, G Chagaluka, Y Chimalizeni, N Kennedy, F Kumwenda, E Nkosi, T Sochera, A Malenga, B Gushu, T Phiri, A Chisale, N Mitole, E Chokani, A Munthali

Imperial College London (Trial Sponsor): K Maitland, TN Williams; Nutritional studies: G Frost, K Walsh
MRC Clinical Trials Unit at UCL, London, UK (oversight): DM Gibb, EC George, M Thomason, D Baptiste, L McCabe, AS Walker

Data Management Systems: A. Ali, K Khamis (Kenya) M Madula, G Abongo (Uganda)

Liverpool School of Tropical Medicine, Liverpool, UK: R Heydermann, I Bates, B Urban

Emma Children’s Hospital AMC Amsterdam, The Netherlands: M Boele von Hensbroek

Independent TRACT Trial Monitors: F Kyomuhendo, S Nakalanzi, J Chabuka, N Mkandawire

Endpoint Review Committee: JA Evans, DM Gibb, F Fitzgerald

Trial Steering Committee: E Molyneux (Chair), I Lubega M Murphy, P Kazembe, J Crawley

Data Monitoring Committee: T Peto (Chair), P Musoke, J Todd, G Mirembe, F Tenu

(b) Trial sites
The TRACT trial recruited children from Uganda (Mbale Regional Referral Hospital, Soroti Regional Hospital, and Mulago Hospital, Kampala) and Malawi (Department/College of Medicine and Malawi-Liverpool-Wellcome Trust Clinical Research Programme, Blantyre).

(c) Further details of randomisation to cotrimoxazole
Cotrimoxazole was provided as dispersible tablets (240mg: trimethoprim 40 mg/sulphamethoxazole 200mg). Dosing followed WHO recommendations for prophylaxis in HIV-infected children, namely 120mg for those aged 2-6 months; 240mg 6 months to 5 years; and 480mg for those >5 years. The dispersible tablets could be taken with water or mixed with feeds.

The cotrimoxazole prophylaxis randomisation was pragmatic in that all children for whom cotrimoxazole prophylaxis should have been prescribed according to WHO or national guidelines (e.g. HIV-infected children) received it regardless of randomisation, and no child in whom it was contraindicated (e.g. known GP6D deficiency according to local testing) received it. The number of children with these conditions was expected to be small (<5%). Such children essentially ignored the cotrimoxazole randomisation on their allocated card; any cotrimoxazole received per guidelines was recorded on case record forms.

At the time of the trial there was no national policy regarding cotrimoxazole supplementation in children with known sickle cell disease (SCD) in Uganda – national guidelines recommended pencillin V for children with SCD under 5 years of age, and malaria prophylaxis with chloroquine. Malawi had no guidelines as sickle cell is rare.

(d) Further details of randomisation to multi-mineral multivitamins (MVMM) and iron/folate

MVMM was provided as Nutromix™[1] (see product specification in appendix), which was specifically designed to meet the recommended nutrient intake (RNI) for children 6-24 months of age with severe anaemia, defined as the daily dietary in−take of a nutrient sufficient to meet the nutrient requirements of nearly all apparently healthy individuals in a specific population group, usually by age and sex. Nutromix has been widely used, for example in studies in children in Ghana[2,3] and Kyrgyz,[4] and in pregnant women.[5]

**Micronutrients**	Per sachet
Vitamin A µg	400
Vitamin D µg	5.0
Vitamin E mg	5.0
Vitamin C mg	30
Thiamine (vitamin B1) mg	0.5
Riboflavin (vitamin B2) mg	0.5
Niacin (vitamin B3) mg	6.0
Vitamin B6 mg	0.5
Vitamin B12 µg	0.9
Folic acid µg	150
Iron mg	10
Zinc mg	4.1
Copper mg	0.56
Selenium µg	17
Iodine µg	90

One sachet was taken daily by the child, mixed into any semi-solid or semi-liquid food, after the food has been prepared and cooled to a temperature acceptable to eat (less than 60^{0}C).

The control group received iron syrup or tablets and folate tablets following WHO guidelines for the management of severe anaemia using local standard-of-care: namely 25mg iron and 100-400micrograms folate daily for children <2 years (depending on the local formulation); and 60mg iron and 400micrograms folate daily for >2 years and <12 years.

The nutritional supplementation, including MVMM randomisation, was pragmatic in that all children for whom these supplements should have been received according to WHO or national guidelines (e.g. those initially admitted with severe malnutrition) received them, and children who should not have received them (e.g. children with severe malnutrition who should not receive iron-containing supplements in the first 7 days of acute rehabilitation) did not receive them. Children with severe malnutrition discharged on ready to use food supplements (RUTF) which contain MVMM essentially ignored the MVMM randomisation on their allocated card, but received their standard post-discharge supplementation within the RUTF which was recorded on case record forms. The number of children with severe malnutrition as their admission diagnosis was expected to be <5%. Children < 6 months who were not weaned (fully breast feed) and therefore could not take MVMM were excluded from the trial.

Recommended nutrient intakes (RNI) with assumed bioavailability of 10%

**Age group**	**MVMM**	**% Iron RNI**	**Iron/Folate**	**% Iron RNI**
0.5-1 years	10mg	108	25mg	269
1-3 years	10mg	172	25 mg	431
4-6 years	10mg	159	60 mg	952
>7 years	10mg	112	60 mg	674

* from WHO/FAO/UNU "Vitamin and mineral requirements in human nutrition, second edition" (page 271) https://apps.who.int/iris/bitstream/handle/10665/42716/9241546123.pdf?ua=1[6]

(e) Further details of allocation concealment

Trial numbers and associated randomized allocations (to all four factorial randomizations) were kept inside opaque sealed envelopes, packed at Kilifi, Kenya, as were separate consecutively numbered packs containing case record

forms and a link to one specific opaque sealed envelope containing a trial number and randomized allocation. The link between pack number and trial number (and hence randomized allocation) was also randomized within blocks. At enrolment, trial staff opened the next consecutively numbered pack, which directed them to a sealed randomization envelope which was within the next 16 envelopes, but was not the next one, ensuring allocation concealment. This method was used to ensure that acutely unwell children could be treated immediately at the emergency ward, without having to rely on a computer or the internet to obtain a randomized allocation.

(f) Further details of endpoint ascertainment and adjudication
SAEs were defined following the International Committee for Harmonisation as events which led to death, were life-threatening, caused or prolonged hospitalization (excluding elective procedures), caused permanent disability, or were other medical conditions or with a real, not hypothetical risk of one of the previous categories.

Deaths (including causes) and trial-drug relatedness were adjudicated by an Endpoint Review Committee (ERC) (two members independent of day to day running of the trial) blind to volume of transfusion and trial drugs received, using the available clinical/laboratory data provided by the sites. They also reviewed all suspected transfusion reactions, suspected respiratory events, suspected neurological events, and allergic reactions.

(g) Sample size calculation from the trial protocol
The sample size calculation was based on 80% power and 2 sided alpha=0.013 to allow for 4 comparisons (factorial randomisations to post-discharge MVMM and/or cotrimoxazole, reported here, plus additional randomisations to 30mls/kg vs 20mls/kg whole blood equivalent transfusion and to any vs no transfusion in those with haemoglobin >4g/dl and no severity signs; uncomplicated severe anaemia). The calculation assumed cumulative mortality at 6 months (primary endpoint for this comparison) would be 28.5% in the complicated severe anaemia stratum, and 21.5% in the uncomplicated severe anaemia stratum, with a 1:1 ratio enrolled in these two subgroups, leading to overall mortality of 25% in those randomised to standard supplementation or no cotrimoxazole prophylaxis. A total of 3162 children provided 80% power to detect a 5% absolute reduction (to 20%), inflated to 3364 assuming 6% lost to follow-up by 6 months. 3954 severe anaemia cases were required to provide >80% power for the transfusion comparisons, so the total sample size enrolled allowed for dilution effects due to a small percentage of children pragmatically not following their allocated MVMM or cotrimoxazole strategy (see (b) and (c) above) and for children dying in hospital or absconding never starting their allocated supplementation.

(h) Statistical methods
The primary analysis was intention-to-treat based on all randomised participants. Randomisation was done at admission, given the relatively low resourced district hospitals in which the trial was undertaken, necessitating the use of envelope-based allocation concealment. Practically, it was not judged feasible to do a second set of randomisations at discharge, and there was also the potential to confuse different sets of randomisation envelopes. Compared to a second randomisation at discharge, the intention-to-treat estimate of intervention effect between groups randomised at admission would have a small amount of dilution bias. A secondary analysis (prespecified in the Statistical Analysis Plan) therefore restricted the population to patients alive at the minimum of discharge or 5 days from randomisation in whom these interventions were neither mandated nor contraindicated (ie excluding HIV-infected children and those with known GP6D deficiency or other contraindications from the cotrimoxazole randomisation, excluding children admitted for severe acute malnutrition, already taking ready-to-use supplementary food or with other contraindications from the supplementation randomisation). Of note, this population is not randomised, because exclusions are based on post-randomisation factors, and therefore this analysis is not formally intention-to-treat. However, the trial was implemented using a factorial design: that is, each randomisation was intrinsically balanced for every other randomisation. Therefore had, for example, more deaths occurred in one transfusion group, balance would still be maintained across both the MVMM and cotrimoxazole randomisations, meaning there is no selection bias in the intention-to-treat comparison of groups as randomised at admission. Any differences in characteristics in the small number of children who died/absconded before discharge (3%) could potentially lead to a small difference in baseline characteristics between those randomised and those who survived to take the MVMM/cotrimoxazole interventions; the impact of this was assessed using standard subgroup analyses (see below),

Time-to-event analyses measured time from randomization, censored at the last ascertainment of vital status if the outcome or any competing events had not occurred. For analyses concerning events at specific time points, surviving children were censored at that time point; specifically for analyses at 28 days, censoring occurred on day 28, for analyses at 90 days censoring occurred on day 90 and for analyses at 180 days censoring occurred on day 183 as this corresponded to 6 months. Analyses of time to readmission measured time from discharge. The primary analyses stratified for randomization stratification factors (centre, stratum) using stratified logrank test and stratified Cox

regression; results from secondary unstratified analyses were very similar (data not shown). Lost-to-follow-up was defined as unknown vital status despite contact tracing by sites.

Analyses of causes of death, and all time-to-event outcomes which did not include all-cause mortality, used competing risks methods. These estimated the probability of the event (analogous to Kaplan-Meier) using cumulative incidence functions, and estimated the effect of randomized group on the sub-distribution hazard corresponding to the cumulative incidence function[7]. For time to haemoglobin recovery and new profound anaemia, death and discharge were considered as competing events. For new profound anaemia, children were only at risk after having one haemoglobin measurement above 4g/dl. These analyses were conducted unstratified, as stratification is not possible with the estimating equation approach used for estimation.

Haemoglobin was compared between randomized groups over time using generalised estimating equations (GEE) (independent correlation structure) with randomized group, adjusting for stratification factors and scheduled visit week as categorical independent variables. The closest measurement to each scheduled visit date within equally spaced windows was used as the measurement at each scheduled visit. Continuous measurements were modelled using change from baseline as the outcome in a normal GEE model, adjusting for baseline value. Haemoglobin below or above thresholds was modelled as a dichotomous outcome in a poisson GEE model to estimate rate ratios. Comparisons at individual timepoints were undertaken using equivalent models without adjusting for repeated measures. Baseline values were those taken at screening.

For each of the primary and secondary outcomes, interactions with the other factorial randomisations (post-discharge cotrimoxazole or multi-vitamins multi-minerals, 20 vs 30 mls/kg transfusion, any vs no transfusion) were tested by including interaction terms in the final model as above.

No adjustment was made for multiple testing.

(i) Subgroup analyses
We pre-specified in the protocol eleven subgroup analyses by the other factorial randomizations (multi-vitamins multi-minerals vs iron and folate, cotrimoxazole vs none, 20 vs 30 mls/kg transfusion, any vs no transfusion; 3 interactions relevant for each randomisation presented here); stratification factors (centre, complicated vs uncomplicated severe anaemia); and six other key factors (previous transfusion ever, fever at screening (temperature >37.5°C), malaria, known sickle cell disease on enrolment or sickle cell disease positive on blood test at admission, HIV (not for the cotrimoxazole vs none comparison as HIV positive children were expected to be prescribed cotrimoxazole following guidelines), and microbiological evidence of sepsis (blood culture or retrospective molecular diagnosis)).

A further five subgroup analyses were pre-specified in the Statistical Analysis Plan, namely by haemoglobinurea, malnutrition, shock (one of weak pulse volume, temperature gradient or capillary refill time), hypothermia, and dehydration.

For continuous factors, we considered both categorisation and natural cubic splines (five knots at the 10th, 25th, 50th, 75th, and 90th centiles) to test for interactions with the main intervention effect of 30mls/kg vs 20mls/kg. All subgroup analyses were conducted unstratified to avoid losing information from small strata with no events in one randomized group.

REFERENCES

1. Zlotkin S, Arthur P, Schauer C, Antwi KY, Yeung G, Piekarz A. Home-fortification with iron and zinc sprinkles or iron sprinkles alone successfully treats anemia in infants and young children. J Nutr 2003;133:1075-80.
2. Adu-Afarwuah S, Lartey A, Brown KH, Zlotkin S, Briend A, Dewey KG. Randomized comparison of 3 types of micronutrient supplements for home fortification of complementary foods in Ghana: effects on growth and motor development. Am J Clin Nutr 2007;86:412-20.
3. Christofides A, Asante KP, Schauer C, Sharieff W, Owusu-Agyei S, Zlotkin S. Multi-micronutrient Sprinkles including a low dose of iron provided as microencapsulated ferrous fumarate improves haematologic indices in anaemic children: a randomized clinical trial. Matern Child Nutr 2006;2:169-80.

4. Lundeen E, Schueth T, Toktobaev N, Zlotkin S, Hyder SM, Houser R. Daily use of Sprinkles micronutrient powder for 2 months reduces anemia among children 6 to 36 months of age in the Kyrgyz Republic: a cluster-randomized trial. Food Nutr Bull 2010;31:446-60.
5. Suchdev PS, De-Regil LM, Walleser S, Vist GE, Pena-Rosas JP. Multiple micronutrient powders for home (point of use) fortification of foods in pregnant women: a systematic review. Geneva: World Health Organization,; 2011.
6. Vitamin and mineral requirements in human nutrition: report of a joint FAO/WHO expert consultation, Bangkok, Thailand, 21–30 September 1998. Geneva: World Health Organization and Food and Agriculture Organization of the United Nations; 2004.
7. Fine JP, Gray RJ. A Proportional Hazards Model for the Subdistribution of a Competing Risk. Journal of the American Statistical Association 1999;94:496-509.

SUPPLEMENTARY FIGURES

Figure S1 Eleven subgroup analyses pre-specified in the protocol for the 180-day mortality primary outcome: MVMM randomisation

Subgroup	MVMM	Iron and folate	MVMM: Iron and folate HR (95% CI)	Interaction p-value
EVER RECEIVED A BLOOD TRANSFUSION (INCLUDING IN THIS ILLNESS)				
Yes	70/675 (10.4%)	70/650 (10.8%)	0.97 (0.70-1.35)	0.94
No	96/1302 (7.4%)	99/1325 (7.5%)	0.98 (0.74-1.30)	
FEVER				
Temperature >37.5°C	62/729 (8.5%)	69/793 (8.7%)	0.98 (0.70-1.38)	0.99
Temperature ≤37.5°C	104/1268 (8.2%)	100/1193 (8.4%)	0.98 (0.74-1.29)	
MALARIA				
Malaria positive	5/497 (1.0%)	5/487 (1.0%)	0.98 (0.28-3.38)	0.71
Malaria negative	79/732 (10.8%)	82/707 (11.6%)	0.93 (0.68-1.27)	
TRANSFUSION VOLUME RANDOMISATION				
30mls/kg	70/803 (8.7%)	84/795 (11.0%)	0.82 (0.60-1.12)	0.22
20mls/kg	70/801 (8.7%)	64/797 (8.0%)	1.09 (0.78-1.53)	
TRANSFUSION TIMING RANDOMISATION				
Immediate	26/393 (6.6%)	21/394 (5.3%)	1.26 (0.71-2.24)	0.68
Control	18/390 (4.6%)	17/388 (4.4%)	1.05 (0.54-2.03)	
COTRIMOXAZOLE RANDOMISATION				
Cotrimoxazole (SoC)	80/1000 (8.0%)	92/994 (9.3%)	0.86 (0.64-1.16)	
No Cotrimoxazole	86/997 (8.6%)	77/992 (7.8%)	1.12 (0.82-1.52)	0.23
SEVERITY STRATA				
Stratum A	122/1214 (10.1%)	131/1204 (10.9%)	0.92 (0.72-1.18)	0.36
Stratum B	44/783 (5.6%)	38/782 (4.9%)	1.16 (0.75-1.79)	
HIV				
Negative	7/721 (1%)	12/719 (1.7%)	1.00 (0.79-1.26)	0.14
Positive	9/58 (15.5%)	13/57 (22.8%)	0.67 (0.28-1.56)	
KNOWN OR PREVIOUSLY UNDIAGNOSED SICKLE CELL DISEASE				
Yes	26/560 (4.6%)	32/529 (6.1%)	0.76 (0.45-1.28)	0.28
No	140/1297 (9.7%)	137/1457 (9.4%)	1.04 (0.82-1.32)	
MICROBIOLOGICAL EVIDENCE OF SEPSIS				
Yes	7/59 (11.9%)	8/66 (12.1%)	0.99 (0.36-2.72)	0.96
No	140/1667 (8.4%)	137/1655 (8.3%)	1.02 (0.80-1.29)	
SITE				
Blantyre	14/233 (6.0%)	12/230 (5.2%)	1.16 (0.54-2.51)	0.70
Mulago	38/472 (8.1%)	45/466 (9.7%)	0.83 (0.54-1.27)	
Soroti	41/535 (7.7%)	45/533 (8.4%)	0.90 (0.59-1.37)	
Mbale	73/757 (9.6%)	67/757 (8.9%)	1.10 (0.79-1.53)	

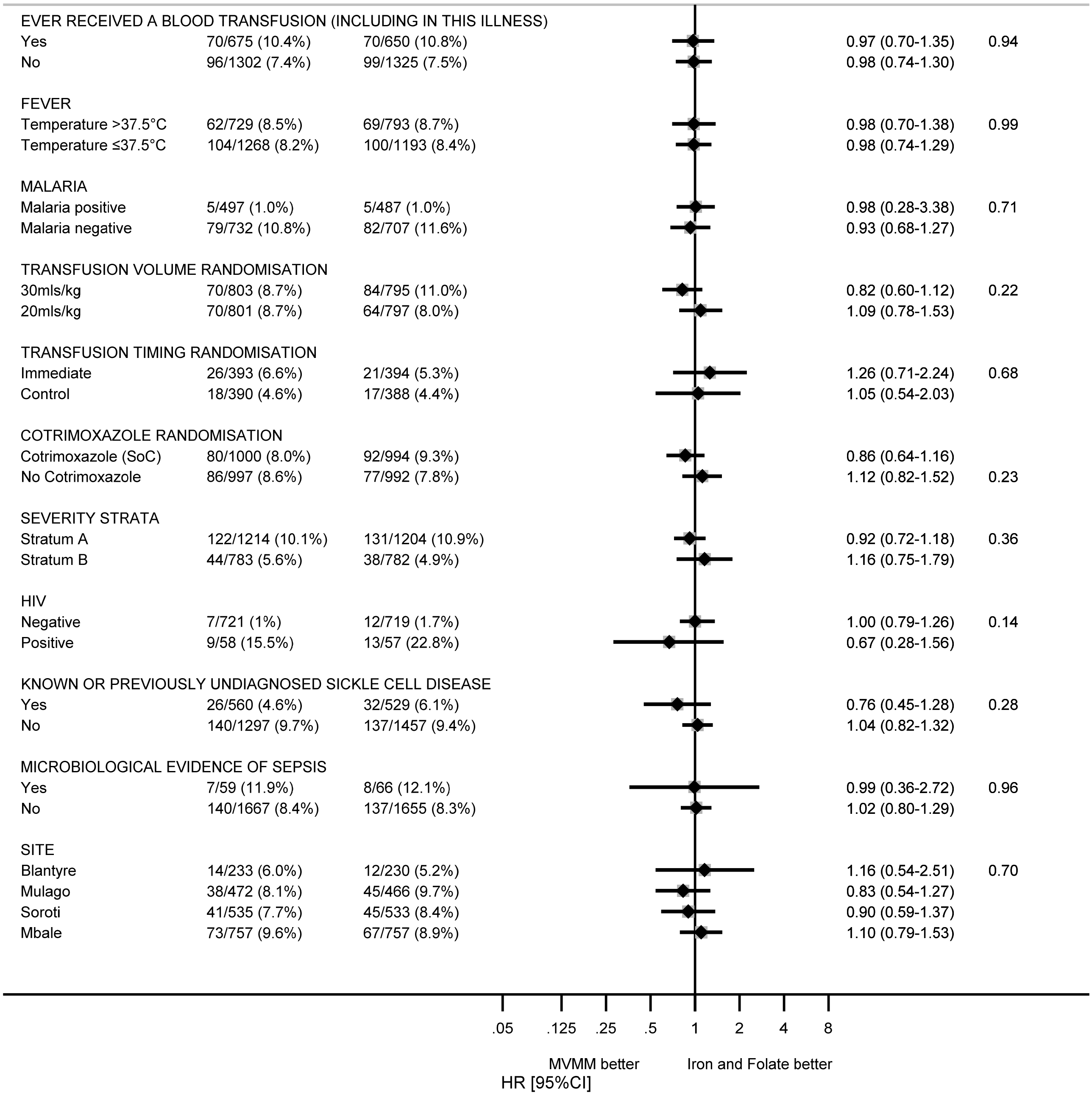

Figure S2 Five subgroup analyses pre-specified in the Statistical Analysis Plan but not the protocol for the 180-day mortality primary outcome: MVMM randomisation

Subgroup	MVMM	Iron and folate	MVMM: Iron and folate HR (95% CI)	Interaction p-value
SHOCK				
Shock at admission	65/572 (11.4%)	76/598 (12.7%)	0.89 (0.64-1.24)	0.41
Not in shock at admission	101/1418 (7.1%)	93/1384 (6.7%)	1.06 (0.80-1.41)	
DEHYDRATION				
Dehydration	21/135 (15.6%)	26/134 (19.4%)	0.76 (0.43-1.35)	0.34
No dehydration	145/1860 (7.8%)	143/1852 (7.7%)	1.01 (0.81-1.28)	
MALNUTRITION				
Well nourished	141/162 (7.6%)	139/1830 (7.6%)	1.00 (0.79-1.26)	0.01*
Under nourished	6/84 (7.1%)	15/97 (15.5%)	0.43 (0.17-1.12)	
Malnourished	8/31 (25.8%)	3/44 (6.8%)	4.40 (1.17-16.62)	
HYPOTHERMIA				
Yes	12/72 (16.7%)	12/75 (16.0%)	1.06 (0.47-2.35)	0.85
No	154/1925 (8.0%)	157/1911 (8.2%)	0.97 (0.78-1.22)	
HAEMOGLOBINURIA				
Yes	33/319 (10.3%)	30/271 (11.1%)	0.94 (0.57-1.54)	0.90
No	133/1678 (7.9%)	139/1715 (8.1%)	0.98 (0.77-1.23)	

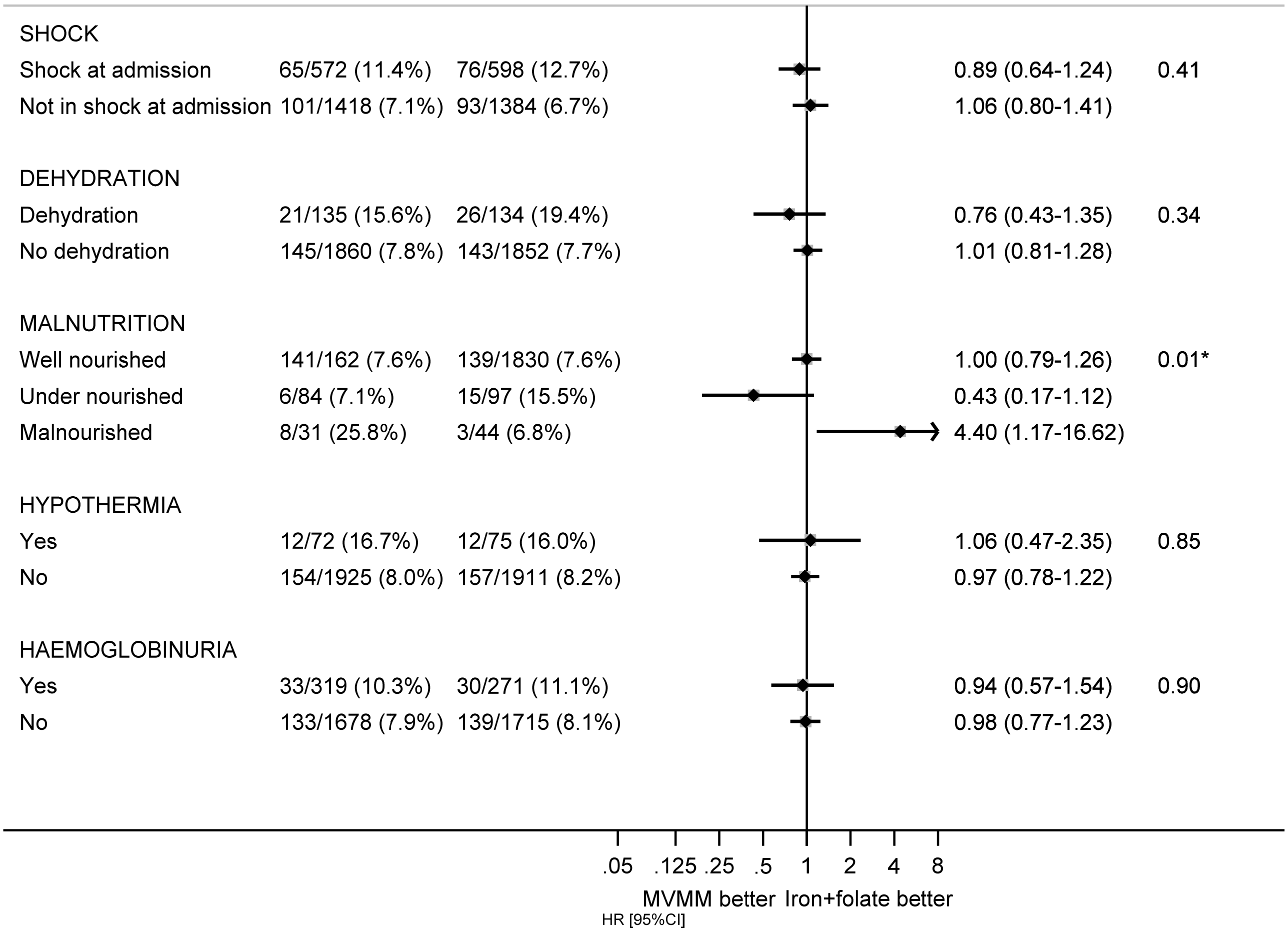

* p=0.57 for interaction between MUAC modelled as a continuous variable and MVMM vs Iron and folate

Figure S3 Ten subgroup analyses pre-specified in the protocol for the 180-day mortality primary outcome: cotrimoxazole randomisation

Subgroup	Cotrimoxazole	No Cotrimoxazole	Cotrimoxazole: No Cotrimoxazole HR (95% CI)	Interaction p-value
EVER RECEIVED A BLOOD TRANSFUSION (INCLUDING IN THIS ILLNESS)				
Yes	75/696 (10.8%)	65/629 (10.3%)	0.97 (0.70-1.35)	0.95
No	97/1289 (7.5%)	98/1338 (7.3%)	1.04 (0.78-1.37)	
FEVER				
Temperature >37.5°C	72/776 (9.3%)	59/746 (7.9%)	1.18 (0.84-1.67)	0.43
Temperature ≤37.5°C	100/1118 (91.8%)	104/1243 (8.4%)	0.99 (0.75-1.30)	
MALARIA				
Malaria positive	84/1288 (6.5%)	90/1249 (7.2%)	0.91 (0.67-1.22)	0.10
Malaria negative	88/702 (12.5%)	73/737 (9.9%)	1.30 (0.95-1.77)	
TRANSFUSION VOLUME RANDOMISATION				
30mls/kg	79/797 (9.9%)	75/801 (9.4%)	1.07 (0.78-1.47)	0.22
20mls/kg	69/801 (8.6%)	65/797 (8.2%)	1.06 (0.75-1.48)	
TRANSFUSION TIMING RANDOMISATION				
Immediate	24/396 (6.1%)	23/368 (5.9%)	1.05 (0.59-1.87)	0.68
Control	21/389 (5.4%)	14/389 (3.6%)	1.50 (0.76-2.96)	
NUTRITIONAL RANDOMISATION				
Iron and folate (SoC)	92/994 (9.3%)	77/992 (7.8%)	1.21 (0.89-1.63)	0.23
Multi-vitamin multi-mineral food additive	80/1000 (8.0%)	86/997 (8.6%)	0.93 (0.69-1.26)	
SEVERITY STRATA				
Stratum A	127/1209 (10.5%)	126/1209 (10.4%)	1.02 (0.80-1.30)	0.48
Stratum B	45/785 (5.7%)	37/780 (4.7%)	1.22 (0.79-1.89)	
KNOWN OR PREVIOUSLY UNDIAGNOSED SICKLE CELL DISEASE				
Yes	29/551 (5.3%)	29/538 (5.4%)	0.98 (0.59-1.64)	0.73
No	143/1443 (9.9%)	134/1451 (9.2%)	1.09 (0.86-1.37)	
MICROBIOLOGICAL EVIDENCE OF SEPSIS				
Yes	7/54 (13.0%)	8/71 (11.3%)	1.18 (0.43-3.24)	0.83
No	143/1665 (8.6%)	134/1657 (8.1%)	1.07 (0.84-1.35)	
SITE				
Blantyre	13/231 (5.6%)	13/232 (5.6%)	0.98 (0.46-2.12)	0.92
Mulago	44/471 (9.3%)	39/467 (8.4%)	1.15 (0.75-1.78)	
Soroti	42/536 (7.8%)	44/532 (8.3%)	0.95 (0.63-1.46)	
Mbale	73/756 (9.7%)	67/758 (8.8%)	1.10 (0.79-1.53)	

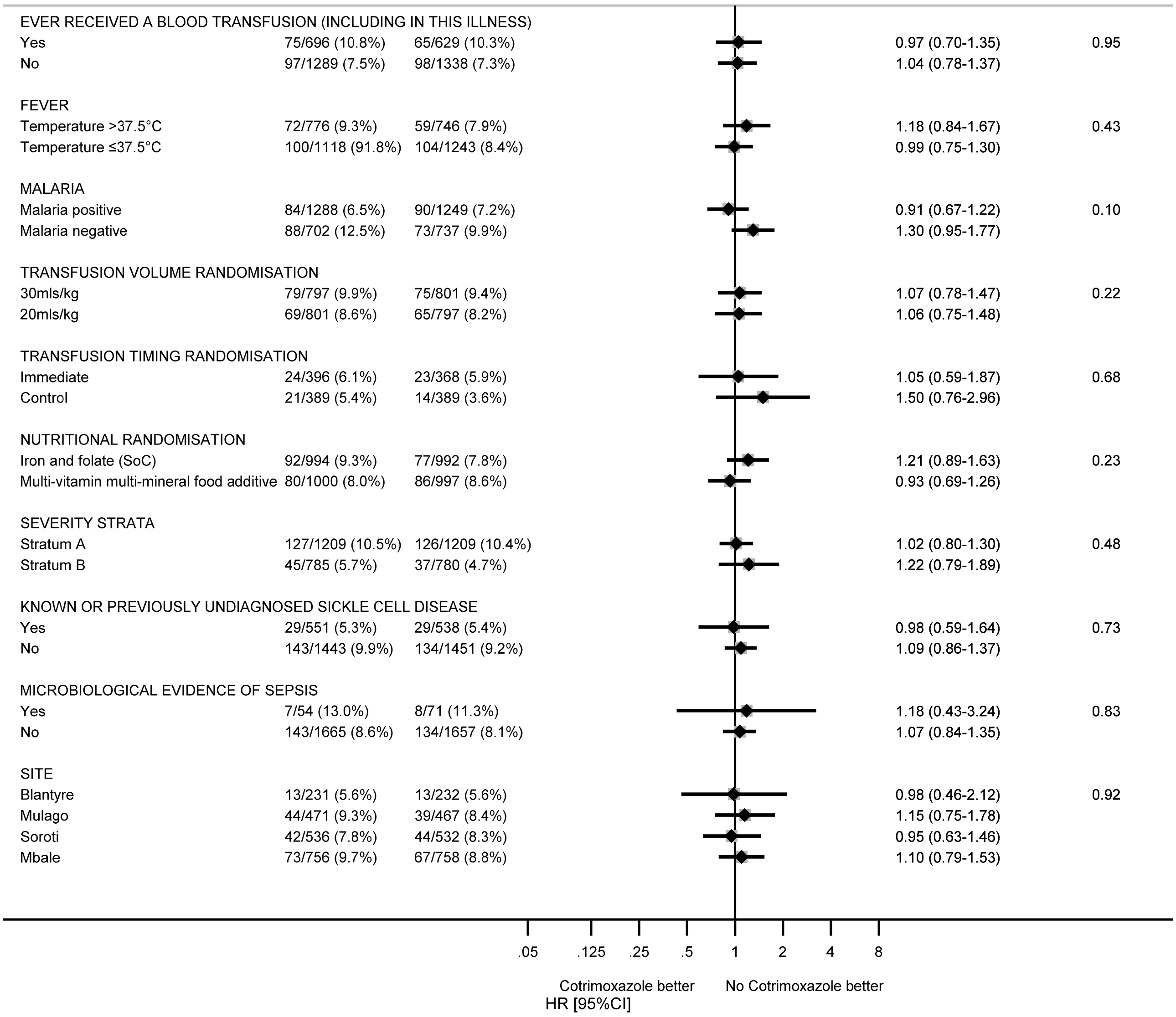

*HIV subgroup not included here as all HIV positive children were expected be prescribed cotrimoxazole as per guidelines and were not expected to follow this randomisation.

Figure S4 Five subgroup analyses pre-specified in the Statistical Analysis Plan but not the protocol for the 180-day mortality primary outcome: cotrimoxazole randomisation

Subgroup	Cotrimoxazole	SoC	Cotrimoxazole: SoC HR (95% CI)	Interaction p-value
SHOCK				
Shock at admission	65/569 (11.4%)	76/601 (12.6%)	0.91 (0.65-1.27)	0.20
Not in shock at admission	107/1421 (7.5%)	87/1381 (6.3%)	1.21 (0.91-1.61)	
DEHYDRATION				
Dehydration	23/123 (18.7%)	24/146 (16.4%)	1.18 (0.67-2.09)	0.72
No dehydration	149/1870 (8.0%)	139/1842 (7.8%)	1.06 (0.84-1.34)	
MALNUTRITION				
Well nourished	145/1854 (7.8%)	135/1838 (7.3%)	1.08 (0.85-1.36)	0.83
Under nourished	10/95 (10.5%)	11/86 (12.8%)	0.83 (0.35-1.97)	
Malnourished	4/28 (14.3%)	7/47 (14.9%)	0.93 (0.27-3.17)	
HYPOTHERMIA				
Yes	11/72 (15.3%)	13/75 (17.3%)	0.8 (0.39-1.97)	0.59
No	161/1922 (8.4%)	150/1914 (7.8%)	1.08 (0.87-1.35)	
HAEMOGLOBINURIA				
Yes	39/308 (12.7%)	24/282 (8.5%)	1.53 (0.92-2.55)	0.11
No	133/2686 (7.9%)	139/1707 (8.1%)	0.97 (0.77-1.24)	

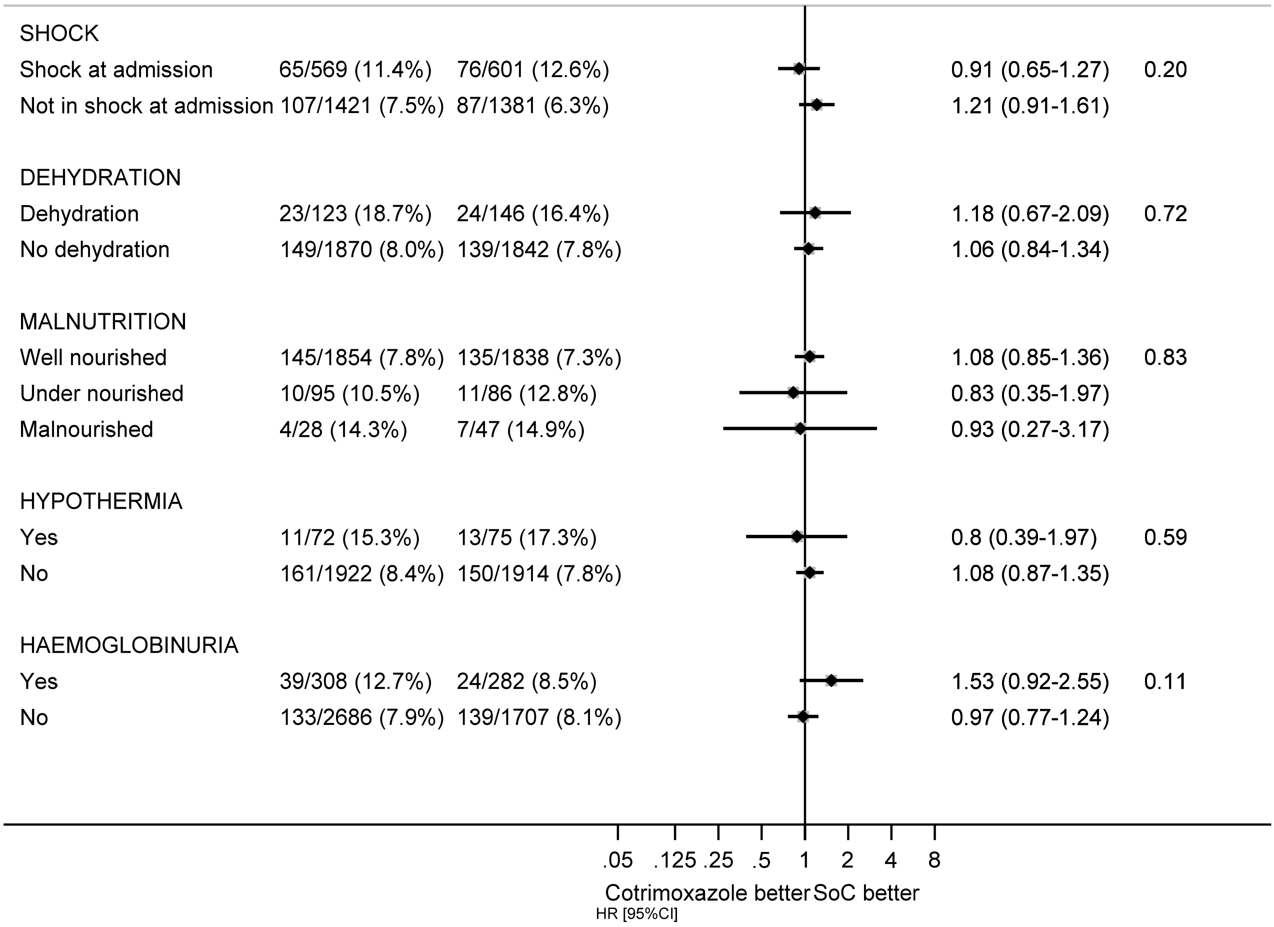

Figure S5 Positive malaria slide

(a) MVMM

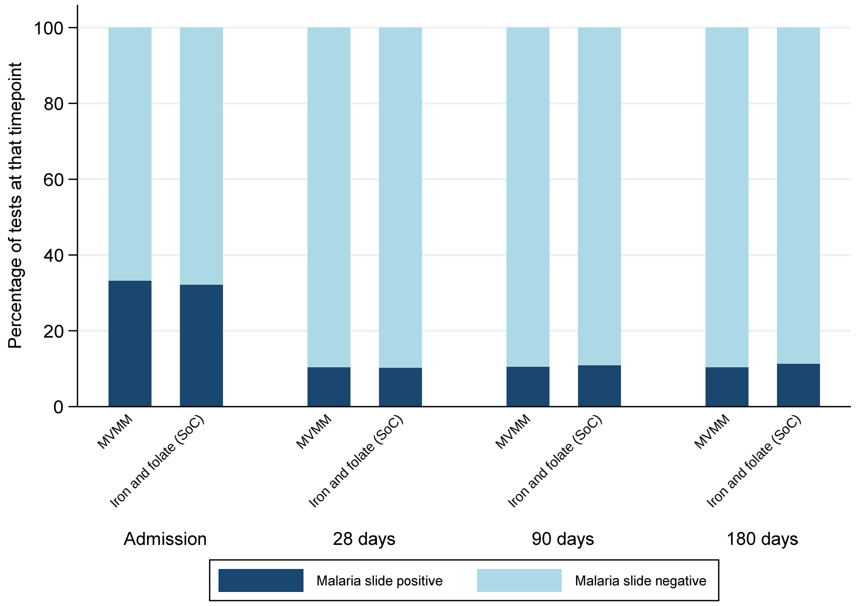

	Admission	28 days	90 days	180 days
MVMM	661/1987 (33%)	192/1837 (10%)	185/1755 (11%)	176/1687 (10%)
Iron and folate	636/1977 (32%)	189/1827 (10%)	191/1740 (11%)	191/1686 (11%)
Total	1297/3964 (33%)	381/3664 (10%)	376/3495 (11%)	367/3373 (11%)
p-value*		0.92	0.68	0.40

(b) Cotrimoxazole

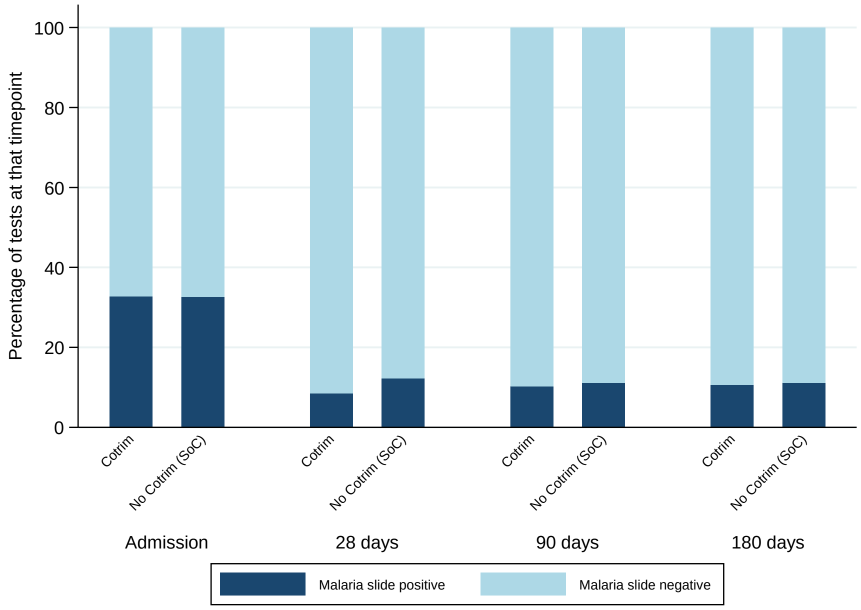

	Admission	28 days	90 days	180 days
Cotrimoxazole	651/1983 (33%)	155/1852 (8%)	180/1743 (10%)	178/1680 (11%)
No Cotrimoxazole	646/1981 (33%)	226/1839 (12%)	196/1752 (11%)	189/1693 (11%)
Total	1297/3964 (33%)	381/3664 (10%)	376/3495 (11%)	367/3373 (11%)
p-value*		<0.001	0.41	0.60

* from Poisson regression

Figure S6 Possible malaria infection*

(a) MVMM

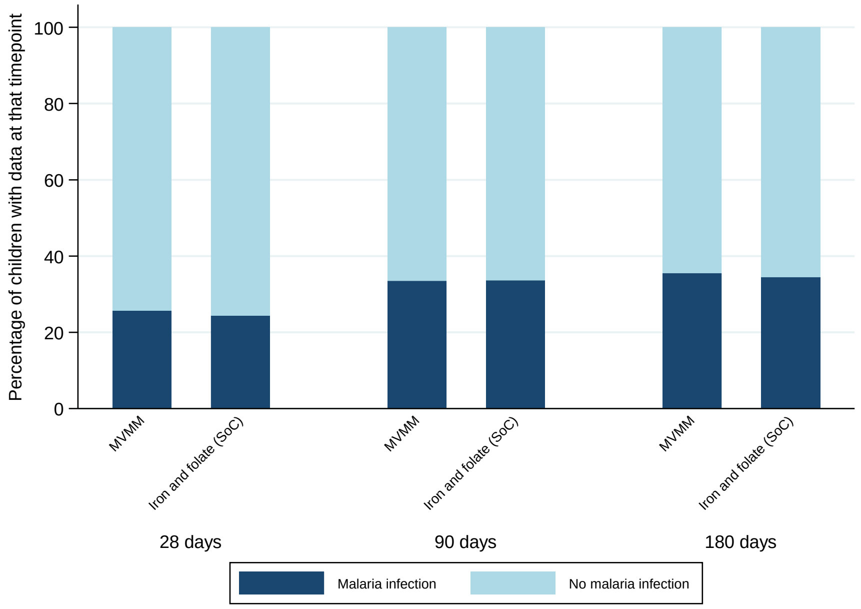

	28 days	90 days	180 days
MVMM	481/1870 (26%)	602/1799 (33%)	615/1733 (35%)
Iron and folate	453/1857 (24%)	601/1790 (34%)	594/1725 (34%)
Total	934/3727 (25%)	1203/3589 (34%)	1209/3458 (35%)
p-value**	0.35	0.94	0.52

(b) Cotrimoxazole

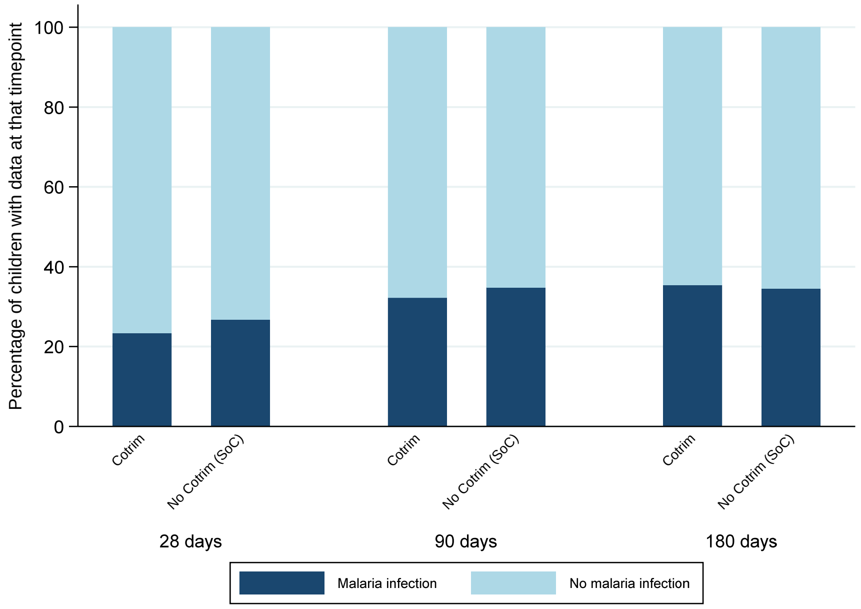

	28 days	90 days	180 days
Cotrimoxazole	434/1859 (23%)	575/1785 (32%)	611/1725 (35%)
No cotrimoxazole	500/1868 (27%)	628/1804 (35%)	598/1733 (35%)
Total	934/3727 (25%)	1203/3589 (33%)	1209/3458 (35%)
p-value**	0.02	0.10	0.57

* Defined as positive malaria slide, or reporting acute febrile illness or having taken antimalarials.

** From Poisson regression.

Figure S7 Possible bacterial infection*

(a) MVMM

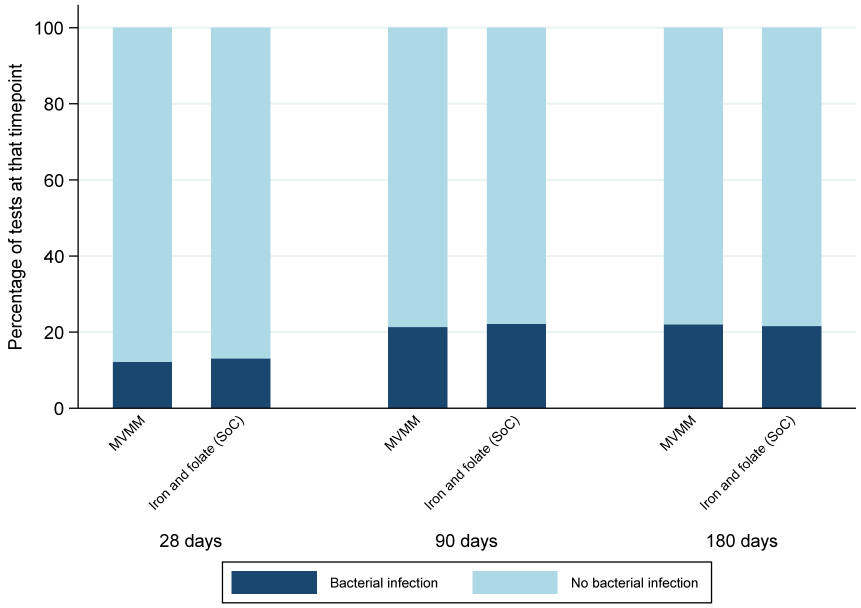

	28 days	90 days	180 days
MVMM	226/1864 (12%)	383/1796 (21%)	379/1724 (22%)
Iron and folate	241/1851 (13%)	396/1786 (22%)	370/1717 (22%)
Total	467/3715 (13%)	779/3582 (22%)	749/3441 (22%)
p-value**	0.41	0.54	0.76

(b) Cotrimoxazole

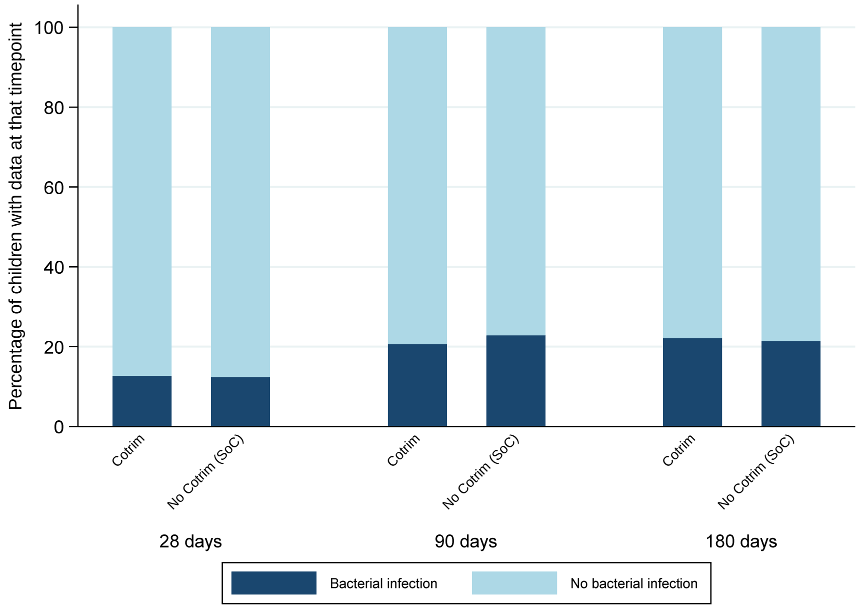

	28 days	90 days	180 days
Cotrimoxazole	236/1853 (13%)	368/1780 (21%)	379/1714 (22%)
No cotrimoxazole	231/1862 (12%)	411/1802 (22%)	370/1727 (21%)
Total	467/3715 (13%)	779/3582 (22%)	749/3441 (22%)
p-value**	0.76	0.12	0.63

* Defined as having had a severe infection since the last visit, or reporting having received antibiotics or having an acute febrile illness.

** From Poisson regression.

Figure S8 Time to new severe bacterial infection

(a) MVMM

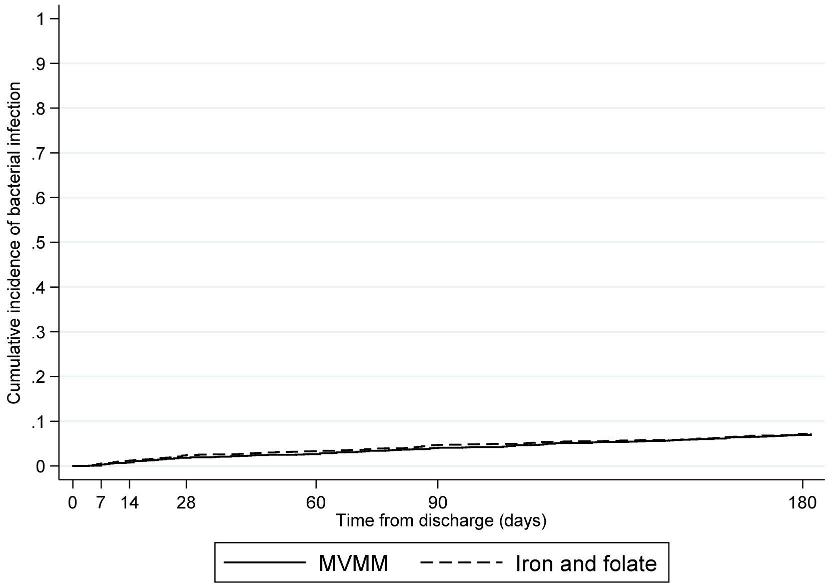

(b) Cotrimoxazole

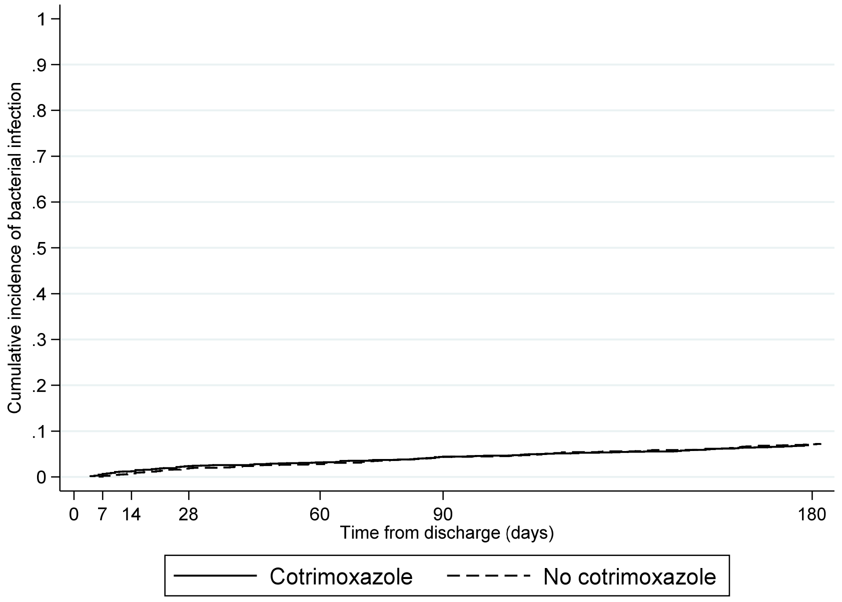

Note: competing risks analysis (with death as the competing risk).

sHR(MVMM:iron/folate)= 0.97 (95%CI 0.77-1.27) p=0.82; 266 events included in analysis (266/3983 (6.7%))

sHR(cotox:no cotox) = 0.96 (95% CI 0.76-1.22) p=0.75

Figure S9 Haemoglobin recovery over 180 days

(a) MVMM randomisation

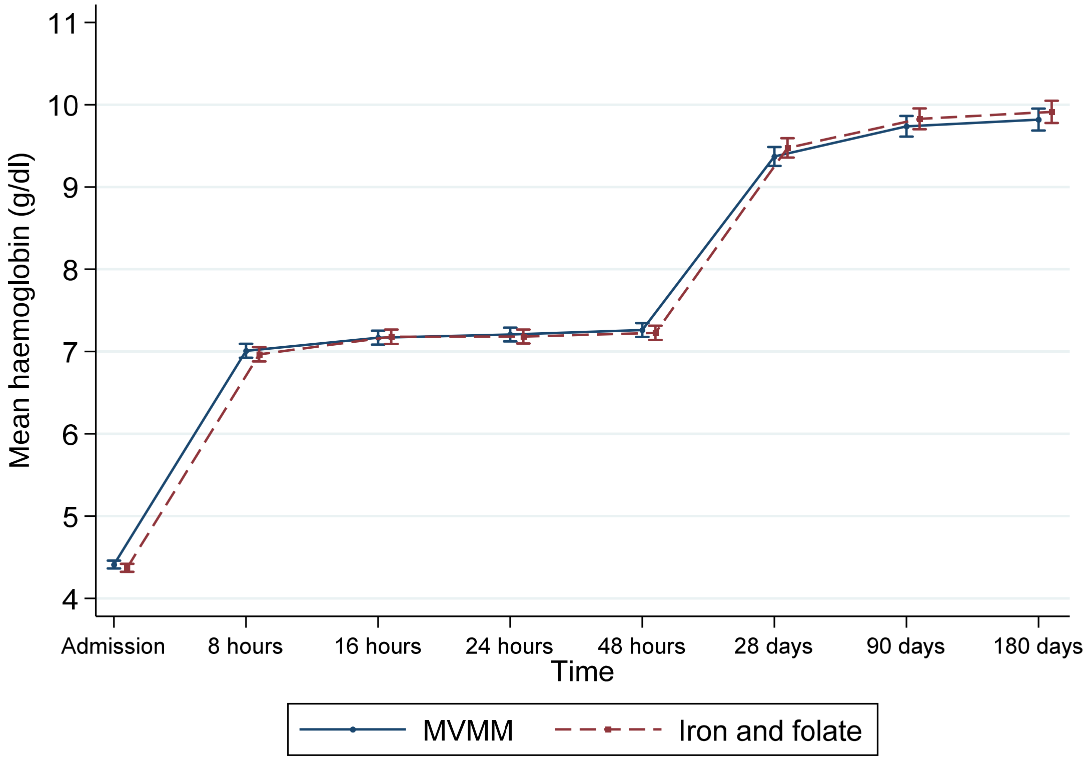

	0 hours	28 days	90 days	180days
N:MVMM	1997	1813	1739	1672
N:Iron and folate	1986	1817	1724	1674
p-value*	-	0.25	0.29	0.28

* comparing change from baseline, adjusting for baseline

(b) Cotrimoxazole randomisation

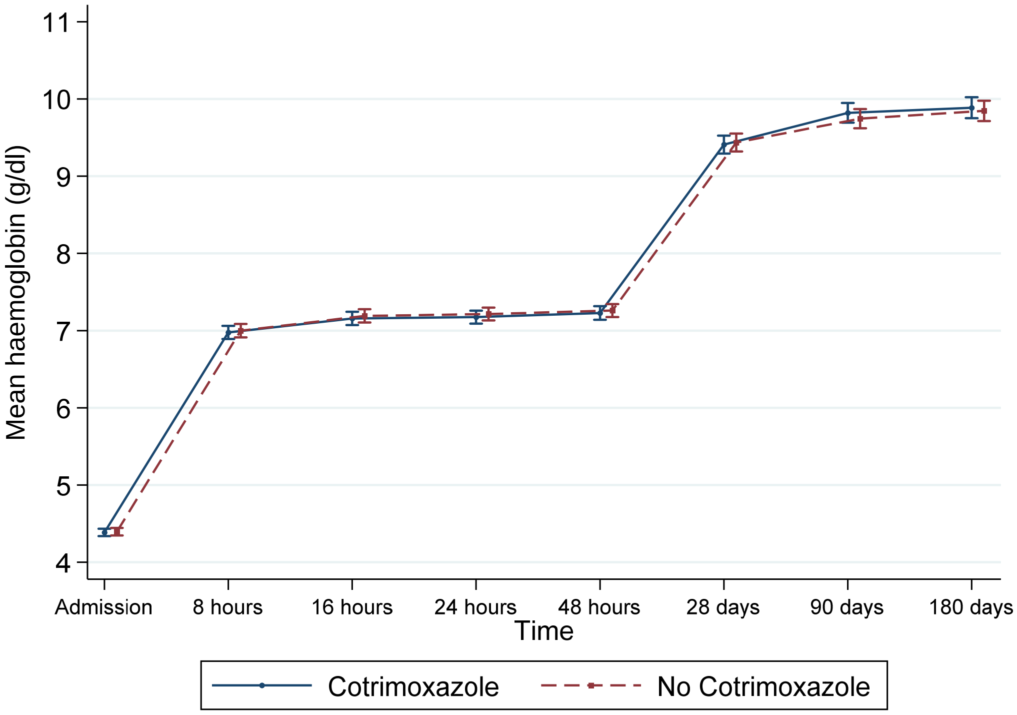

	0 hours	28 days	90 days	180days
N: Cotrimoxazole	1994	1814	1720	1665
N: No cotrimoxazole	1989	1816	1743	1681
p-value*		0.75	0.45	0.67

* comparing change from baseline, adjusting for baseline

Figure S10 Impact of MVMM on haemoglobin recovery over 180 days by age group (with varying dosage of iron)

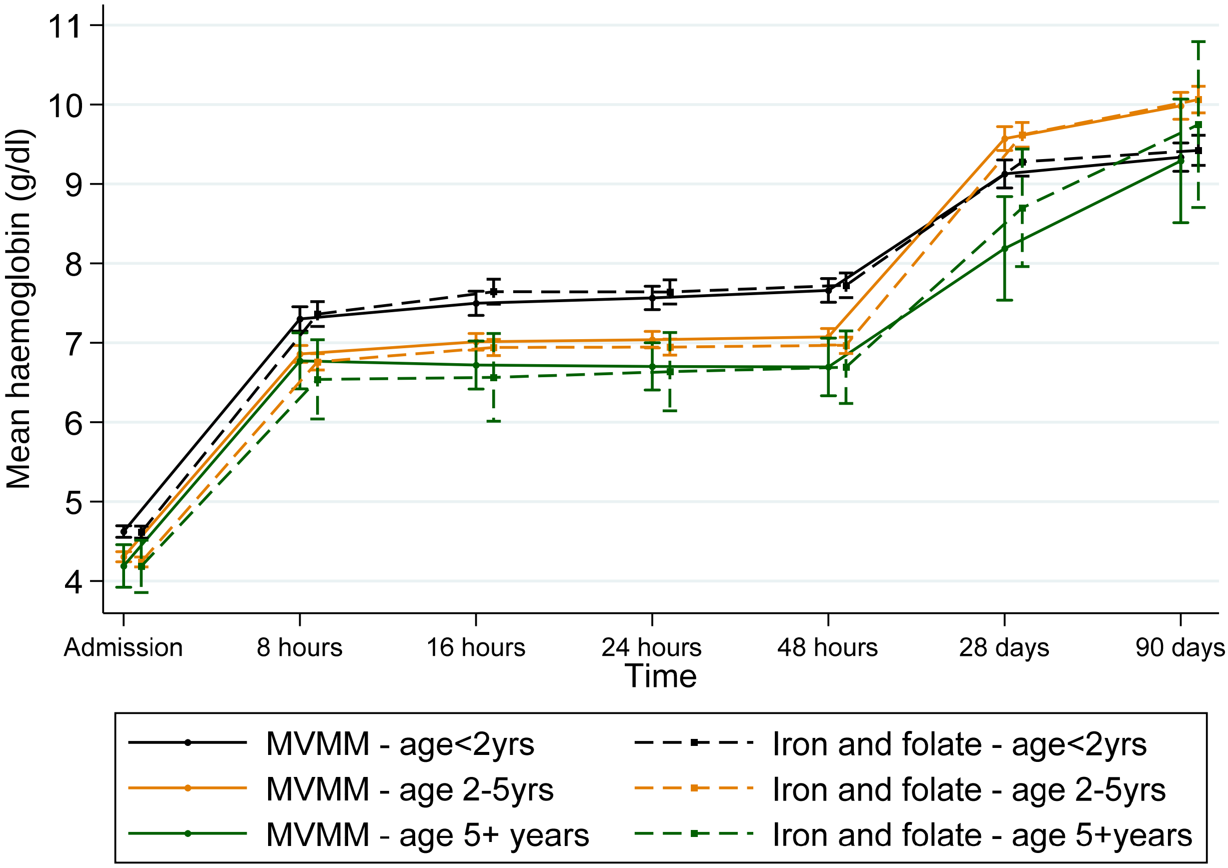

Note: children <2 years (1400, 35%) received 10mg vs 25mg iron if randomised to MVMM vs iron/folate respectively, whereas those >2 years received 10mg vs 60mg iron respectively.

Figure S11 Impact of MVMM on haemoglobin recovery post-discharge by receipt of blood transfusion in TRACT B only

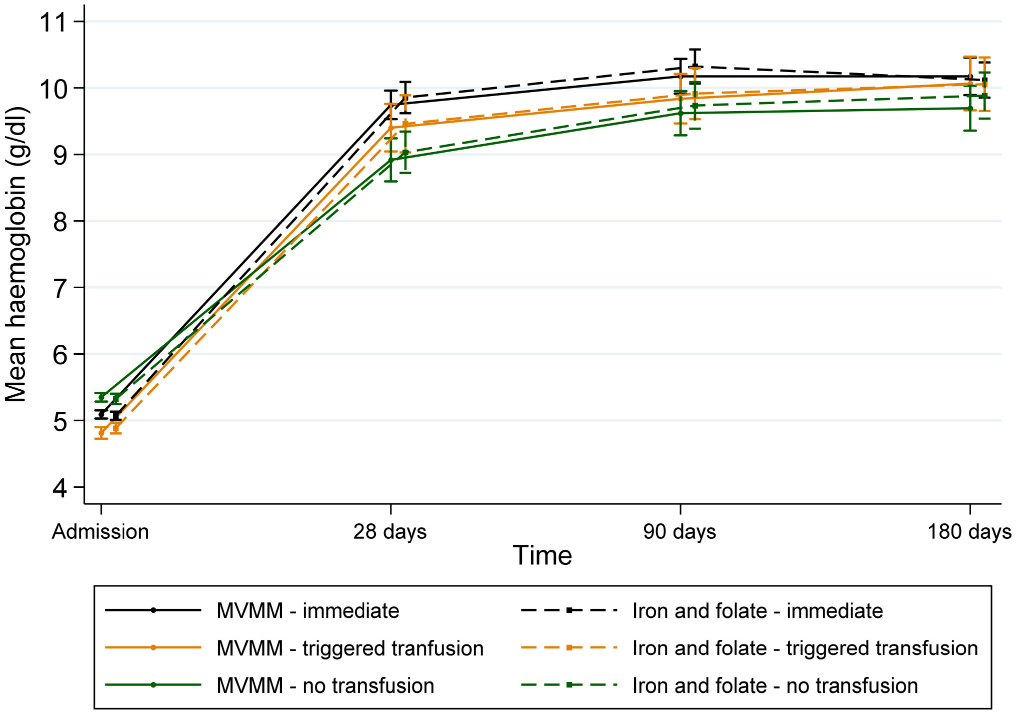

Note: restricted to children with haemoglobin 4-6g/dl and no severity signs enrolled in TRACT B who were randomised to immediate vs triggered transfusion. Triggered transfusion group split (non-randomised) into those who did vs did not receive a transfusion triggered by new severity signs.

There was no evidence of a differential effect of MVMM vs iron/folate according to immediate vs triggered received vs triggered no transfusion received groups (ie no evidence of interaction) at 28 days (p=0.83), 90 days (p=0.75) or 180 days (p=0.63) adjusting for predictors of receiving a transfusion (baseline haemoglobin, age, heart rate and centre).

Figure S12 Impact of MVMM on malaria by age group (varying dosage of iron)

(a) Positive malaria slide

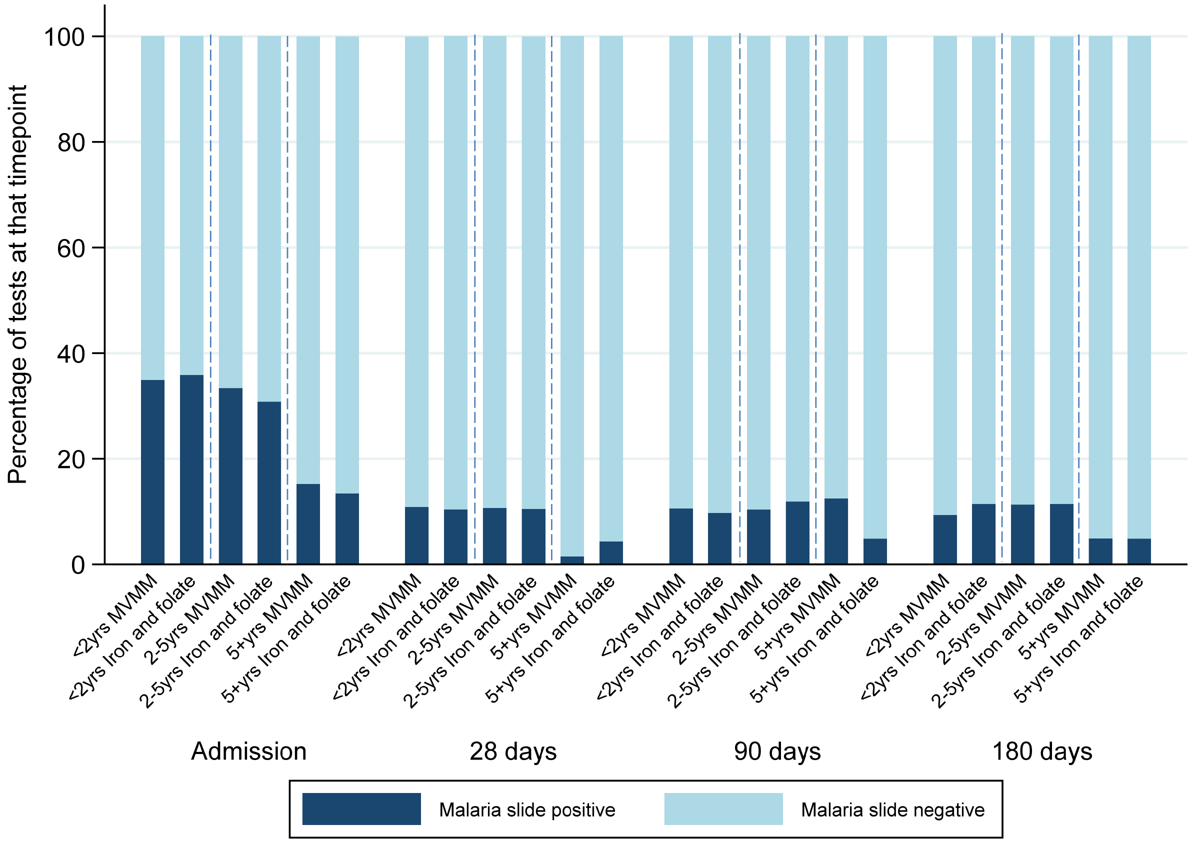

	Admission			28 days			90 days			180 days		
Age group	<2 years	2-5 years	5+ years	<2 years	2-5years	5+ years	< 2 years	2-5 years	5+ years	<2 years	2-5 years	5+ year
MVMM	241/690 (35%)	409/1225 (33%)	11/72 (15%)	68/623 (11%)	123/1149 (11%)	1/65 (2%)	63/593 (11%)	114/1098 (10%)	8/64 (13%)	53/567 (9%)	120/1059 (11%)	3/61 (5%)
Iron and folate	254/708 (36%)	375/1217 (31%)	7/52 (13%)	67/644 (10%)	120/1137 (11%)	2/46 (4%)	60/616 (10%)	129/1083 (12%)	2/41 (5%)	68/592 (11%)	121/1053 (11%)	2/41 (5%)
Total	495/1398 (35%)	784/2442 (32%)	18/124 (15%)	135/1267 (10%)	243/2286 (11%)	3/111 (3%)	123/1209 (10%)	243/2181 (11%)	10/105 (10%)	121/1159 (10%)	221/2112 (11%)	5/102 (5%)
p-value**				0.77	0.91	0.39	0.61	0.26	0.22	0.24	0.91	0.99
Heterogeneity				0.67			0.24			0.66		

(b) Possible malaria infection*

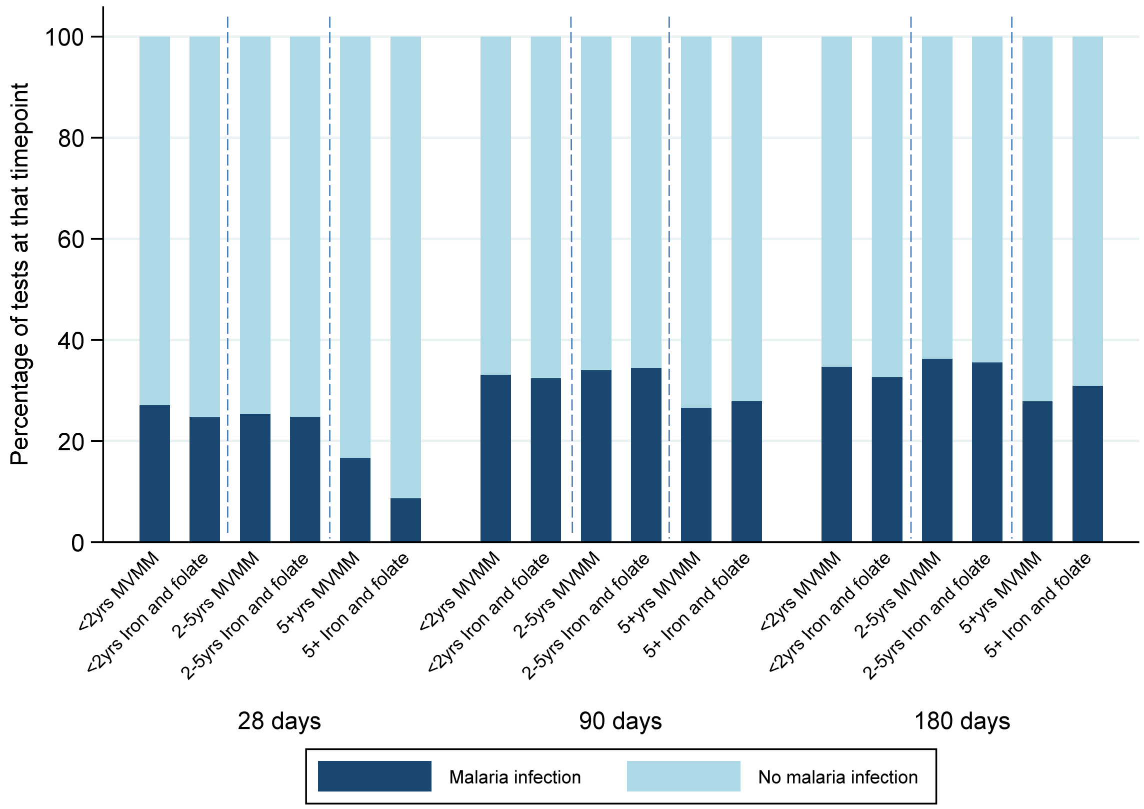

	28 days			90 days			180 days		
Age group	<2 years	2-5years	5+ years	< 2 years	2-5 years	5+ years	<2 years	2-5 years	5+ years
MVMM	172/634 (27%)	298/1170 (25%)	11/66 (17%)	203/613 (33%)	382/1122 (34%)	17/64 (27%)	203/584 (35%)	395/1088 (36%)	17/61 (28%)
Iron and folate	162/653 (25%)	287/1158 (25%)	4/46 (9%)	206/635 (32%)	383/1112 (34%)	12/43 (28%)	199/609 (33%)	382/1074 (36%)	13/42 (31%)
Total	334/1287 (26%)	585/2328 (25%)	15/112 (13%)	409/1248 (33%)	765/2234 (34%)	29/107 (27%)	402/1193 (34%)	777/2162 (36%)	30/103 (29%)
p-value**	0.34	0.70	0.24	0.80	0.84	0.88	0.45	0.72	0.74
Heterogeneity p	0.49			0.74			0.56		

* Defined as positive malaria slide, or reporting acute febrile illness or having taken antimalarials.
** From Poisson regression.

Note: children <2 years (1400, 35%) received 10mg vs 25mg iron if randomised to MVMM vs iron/folate respectively, whereas those >2 years received 10mg vs 60mg iron respectively.

SUPPLEMENTARY TABLES

Table S1 Additional baseline characteristics of randomised children

	MVMM (N=1997)	Iron+folate (N=1986)	Cotrimoxazole (N=1994)	No cotrimoxazole (N=1989)	Total (N=3983)
Randomised within 24h of admission	1917 (96%)	1896 (96%)	1904 (96%)	1909 (96%)	3813 (96%)
Systolic blood pressure (mmHg)	91 (83,99)	92 (84,99)	92 (84,99)	91 (84,99)	91 (84,99)
Diastolic blood pressure (mmHg)	54 (48,62)	55 (48,62)	55 (48,62)	54 (48,62)	54 (48,62)
Capillary refill time (seconds)	2 (1,2)	2 (1,2)	2 (1,2)	2 (1,2)	2 (1,2)
Temperature gradient (shin to thigh)	183 (9%)	181 (9%)	187 (9%)	177 (9%)	364 (9%)
Weak radial pulse	45 (2%)	64 (3%)	53 (3%)	56 (3%)	109 (3%)
Previous blood transfusion in this illness	31 (2%)	40 (2%)	38 (2%)	33 (2%)	71 (2%)
Blood transfusion ever	665 (34%)	644 (33%)	686 (35%)	623 (32%)	1309 (33%)
Number of transfusions ever					
One transfusion	253 (13%)	271 (14%)	250 (13%)	274 (14%)	524 (13%)
Two transfusions	150 (8%)	124 (6%)	151 (8%)	123 (6%)	274 (7%)
Three or four transfusions	125 (6%)	117 (6%)	135 (7%)	107 (5%)	242 (7%)
Five or more transfusions	133 (7%)	129 (7%)	144 (7%)	118 (6%)	262 (7%)
Impaired consciousness	361 (18%)	397 (20%)	371 (19%)	387 (19%)	758 (19%)
Respiratory distress	419 (21%)	441 (22%)	422 (21%)	438 (22%)	860 (22%)
Haemoglobinuria	319 (16%)	271 (14%)	308 (15%)	282 (14%)	590 (15%)
Profound anaemia (<4 g/dl)	663 (33%)	679 (34%)	683 (34%)	659 (33%)	1342 (34%)
Number of severity features					
One feature	645 (32%)	639 (32%)	630 (32%)	654 (33%)	1284 (32%)
Two features	376 (19%)	375 (19%)	385 (19%)	366 (18%)	751 (19%)
Three features	164 (8%)	157 (8%)	166 (8%)	155 (8%)	321 (8%)
Four features	28 (1%)	30 (2%)	27 (1%)	31 (2%)	58 (1%)
Five features	4 (0%)	3 (0%)	2 (0%)	5 (0%)	7 (0%)

Note: showing numbers (%) or median and interquartile ranges (IQR).

Table S2 Prescription and adherence
(a) MVMM vs iron/folate

	MVMM (N=1977)	Iron/folate (N=1986)	Total (N=3983)
At initial prescription			
Receiving MVMM	1901 (95%)	0 (0%)	1901 (48%)
Receiving iron and folate	0 (0%)	1901 (96%)	1911 (48%)
Prescribed at discharge (%)	1515 (76%)	1499 (75%)	3014 (76%)
Prescribed before discharge (%)	386 (19%)	412 (21%)	798 (20%)
Receiving plumpynut (ready-to-use therapeutic food)	19 (1%)	14 (1%)	19 (0%)
Died or absconded before prescription	69 (3%)	64 (3%)	133 (3%)
Not prescribed as contraindicated	8 (0%)	7 (0%)	19 (0%)
Median (IQR) [range] days until prescription	4 (3,5) [1,49]	4 (3,5) [1,43]	4 (3,5) [1,49]
At 28 days			
Receiving MVMM	1812 (91%)	0 (0%)	1812 (45%)
Receiving iron and folate	0 (0%)	1817 (91%)	1817 (46%)
Not receiving - stopped at/before visit	26 (1%)	7 (0%)	33 (1%)
Died/lost to follow-up before this visit	90 (5%)	94 (5%)	184 (5%)
Missed visit	47 (2%)	42 (2%)	89 (2%)
Had visit but no adherence details	22 (1%)	26 (1%)	48 (1%)
Number of children reported by carer to have missed any doses (% receiving since last visit)	625 (34%)	460 (25%)	1085 (30%)
Timing of missed doses* (% receiving since last visit)			
Yesterday	93 (5%)	96 (5%)	189 (5%)
2-7 days ago	256 (13%)	253 (13%)	509 (13%)
8-14 days ago	276 (14%)	181 (9%)	457 (12%)
15-28 days ago	218 (11%)	135 (7%)	353 (9%)
Number of children returning any tablets/sachets (% receiving since last visit)	702 (39%)	380 (21%)	1082 (30%)
Median (IQR) tablets/sachets returned	3 (1,5)	3 (2,5)	3 (1,5)
Returned tablets/sachets (% receiving since last visit)			
None	857 (47%)	1038 (57%)	1895 (52%)
Up to 7 tablets	590 (33%)	328 (18%)	918 (25%)
8 or more tablets	112 (6%)	52 (3%)	164 (5%)
No tablet/sachet count	253 (14%)	399 (22%)	652 (18%)
At 90 days			
Receiving MVMM until this visit	1726 (86%)	0 (0%)	1726 (43%)
Receiving iron and folate until this visit	1 (0%)	1716 (86%)	1717 (43%)
Not receiving - stopped earlier	31 (2%)	33 (2%)	64 (2%)
Died or LTFU before this visit	154 (8%)	152 (8%)	306 (8%)
Missed visit	50 (3%)	50 (3%)	100 (3%)
Had visit but no adherence details	35 (2%)	35 (2%)	70 (2%)
Number of children reported by carer to have missed any doses (% receiving since last visit)	687 (39%)	517 (30%)	1204 (34%)
Timing of missed doses* (% receiving since last visit)			
Yesterday	91 (5%)	124 (6%)	215 (6%)

	MVMM (N=1977)	Iron/folate (N=1986)	Total (N=3983)
2-7 days ago	260 (13%)	261 (14%)	521 (13%)
8-14 days ago	279 (14%)	177 (9%)	456 (12%)
15-28 days ago	242 (12%)	156 (8%)	398 (10%)
>28 days ago	186 (10%)	102 (5%)	288 (7%)
Number of children returning any tablets/sachets (% receiving since last visit)	697 (40%)	565 (32%)	1262 (36%)
Median (IQR) tablets/sachets returned	5 (3,10)	4 (2,8)	5 (3,9)
Returned tablets/sachets (% receiving since last visit)			
None	827 (47%)	875 (50%)	1702 (49%)
Up to 7 tablets	454 (26%)	396 (23%)	850 (24%)
8 or more tablets	243 (14%)	169 (10%)	412 (12%)
No tablet/sachet count	218 (13%)	299 (17%)	517 (15%)

* not mutually exclusive, ie carers could report missing doses in each of the time windows

(b) cotrimoxazole

	Cotrimoxazole (N=1994)	No cotrimoxazole (N=1989)	Total (N=3983)
At initial prescription			
Receiving cotrimoxazole	1922 (96%)	56 (3%)*	1928 (48%)
Prescribed at discharge (%)	1513 (76%)	0 (0%)	1513 (38%)
Prescribed before discharge (%)	408 (20%)	56 (3%)	413 (10%)
Not receiving cotrimoxazole	5 (0%)	1933 (100%)	1988 (50%)
Not receiving - contraindicated	6 (0%)	0 (0%)	6 (0%)
Died or absconded before prescription	61 (3%)	0 (0%)	61 (2%)
Median (IQR) [range] days until prescription	4 (3,5) [1,70]	4 (3,5) [1,49]	4 (3,5) [1,70]
At 28 days			
Receiving cotrimoxazole	1798 (90%)	47 (2%)*	1802 (45%)
Not receiving cotrimoxazole	0 (0%)	1791 (90%)	1834 (46%)
Not receiving - stopped at/before visit	28 (1%)	0 (0%)	28 (1%)
Died or LTFU before visit	102 (5%)	82 (4%)	184 (5%)
Missed visit	42 (2%)	47 (2%)	89 (2%)
Had visit but no adherence details	24 (1%)	22 (1%)	46 (1%)
Number of children reported by carer to have missed any doses (% receiving since last visit)	466 (26%)	-	-
Timing of missed doses* (% receiving since last visit)			
Yesterday	88 (5%)	-	-
2-7 days ago	234 (12%)	-	-
8-14 days ago	180 (10%)	-	-
15-28 days ago	144 (8%)	-	-
Number of children returning any tablets (% receiving since last visit)	246 (16%)	-	-
Median (IQR) tablets returned	3 (1,6)	-	-
Returned tablets/sachets (% receiving since last visit)			-
None	980 (62%)	-	

	Cotrimoxazole (N=1994)	No cotrimoxazole (N=1989)	Total (N=3983)
Up to 7 tablets	212 (13%)	-	-
8 or more tablets	34 (2%)	-	-
No tablet/sachet count	352 (22%)	-	-
At 90 days			
Received cotrimoxazole until this visit	1693 (85%)	49 (2%)*	1698 (43%)
Not receiving cotrimoxazole	0 (0%)	1713 (86%)	1757 (44%)
Not receiving - stopped earlier	54 (3%)	0 (0%)	54 (1%)
Died or LTFU before visit	166 (8%)	140 (7%)	306 (8%)
Missed visit	50 (3%)	50 (3%)	100 (3%)
Had visit but no adherence details	31 (2%)	37 (2%)	68 (2%)
Number of children reported by carer to have missed any doses (% receiving since last visit)	523 (31%)	-	-
Timing of missed doses* (% receiving since last visit)			
Yesterday	100 (5%)	-	-
2-7 days ago	220 (12%)	-	-
8-14 days ago	205 (11%)	-	-
15-28 days ago	165 (9%)	-	-
>28 days ago	111 (6%)	-	-
Number of children returning any tablets (% receiving since last visit)	447 (27%)	-	-
Median (IQR) tablets returned	4 (2,8)	-	-
Returned tablets/sachets (% receiving since last visit)			
None	955 (58%)	-	-
Up to 7 tablets	317 (19%)	-	-
8 or more tablets	130 (8%)	-	-
No tablet/sachet count	236 (14%)	-	-

* 55/56 had HIV and so received cotrimoxazole according to guidelines. 3 HIV positive children were not found to have a prescription for cotrimoxazole in their notes, 1/3 had a reported allergy.
** not mutually exclusive, ie carers could report missing doses in each of the time windows

Table S3 Causes of death as adjudicated by the Endpoint Review Committee

ERC Adjudication	MVMM and cotrimoxazole N=80	MVMM only N=86	Iron and folate and cotrimoxazole N=92	Iron and folate N=77	Total N=355
Unknown	33	47	55	34	169
Haematological	18	10	12	13	53
Anaemia with clinical symptoms	15	9	10	9	43
Pancytopenia/bone marrow depression	3	1	2	3	9
Sickle cell crisis	0	0	0	1	1
Specific infections	12	13	16	17	58
P falciparum malaria	8	8	12	10	38
Cerebral Malaria	0	1	0	0	1
Meningitis lumbar puncture diagnosed – no organism (no culture)	0	1	1	0	2
Meningitis no lumbar puncture	0	0	1	0	1
Other gram negative sepsis	1	1	0	1	3
Presumed septicaemia/bacteraemia - no organism	0	1	0	0	1
Presumed septicaemia/bacteraemia - not investigated	3	1	2	5	11
Tuberculosis - disseminated/miliary	0	0	0	1	1
Lower respiratory tract	7	3	6	7	23
Empyema	1	0	0	0	1
Pneumonia no organism identified/aspiration pneumonia	6	3	6	7	22
Tumours	3	8	1	3	15
Burkitts lymphoma	0	1	0	1	2
Leukaemia	2	6	0	2	10
Other solid tumour	1	1	1	0	3
Renal or hepatic	1	2	1	0	4
Nephrotic syndrome	0	1	0	0	1
Renal failure - acute	0	0	1	0	1
Renal failure - chronic	0	1	0	0	1
Hepatitis cause unknown	1	0	0	0	1
Gastrointestinal	2	2	1	0	4
Acute abdomen	0	0	1	0	1
Haematemesis	2	0	0	0	2
Acute diarrhoea not investigated	0	2	0	0	2
Other					5
Congestive cardiac failure	1	0	0	0	1
Dehydration	0	0	0	1	1
Encephalitis – presumed infectious	1	0	0	1	2
Encephalopathy – unspecified	0	0	0	1	1
Traumatic	1	1	0	0	2
TRALI (transfusion related acute lung injury)	1	0	0	0	1

Table S4 All SAEs

	MVMM and Cotrimoxazole (N=327)	MVMM only (N=343)	Iron and folate and Cotrimoxazole (N=346)	Iron and folate (N=335)	Total (N=670)
death not otherwise specified	36	34	42	39	70
anaemia	73	58	71	64	131
anaemia+arthralgia+arthitis	0	0	1	0	0
anaemia+asthma	0	0	0	1	0
anaemia+bronchiolitis	0	1	0	1	1
anaemia+cardiac failure+renal failure	0	0	1	0	0
anaemia+epigastric pain+vomiting	0	1	0	0	1
anaemia+epistaxis	1	0	0	0	1
anaemia+fatigue	0	1	0	0	1
anaemia+fatigue+non-hodgkins lymphoma	0	0	1	0	0
anaemia+intestinal obstruction	0	1	0	0	1
anaemia+lactic acidosis	1	1	0	0	2
anaemia+leukaemia	0	1	1	1	1
anaemia+leukaemia+lymphadenopathy	1	0	0	0	1
anaemia+leukaemia+thrombocytopenia	0	2	0	0	2
anaemia+parasthesia	0	0	1	0	0
anaemia+pericarditis	1	0	0	0	1
anaemia+respiratory failure	0	1	0	0	1
anaemia+shock	0	0	0	1	0
anaemia+sickle cell crisis	4	4	5	6	8
anaemia+splenomegaly	0	1	0	1	1
anaemia+thrombocytopenia	1	0	1	0	1
anaemia+thrombocytopenia+raised lfts	1	0	0	0	1
anaemia+DUS	12	8	19	9	20
Anaemia+DUS+hypoglycaemia	0	1	0	0	1
anaemia+DUS+injury	1	0	0	0	1
anaemia+DUS+lactic acidosis	0	0	0	1	0
anaemia+fever+DUS	0	0	1	0	0
anaemia+malaria	23	25	26	30	48
anaemia+malaria+convulsions	1	0	0	0	1
anaemia+malaria+DUS	1	8	4	6	9
anaemia+malaria+DUS+hypoglycaemia	1	0	0	0	1
anaemia+malaria+lactic acidosis	0	1	0	0	1
anaemia+malaria+malnutrition	0	0	0	1	0
anaemia+malaria+sickle cell crisis	0	1	1	1	1
anaemia+malaria+vomiting	0	0	0	1	0
anaemia+malaria+DUS+sepsis	0	1	0	1	1
anaemia+malaria+gastroenteritis	0	2	0	1	2
anaemia+malaria+gastroenteritis+sepsis	0	0	0	1	0

	MVMM and Cotrimoxazole (N=327)	MVMM only (N=343)	Iron and folate and Cotrimoxazole (N=346)	Iron and folate (N=335)	Total (N=670)
anaemia+malaria+LRTI/pneumonia	1	2	0	2	3
anaemia+malaria+LRTI/pneumonia+cardiac failure	0	0	0	1	0
anaemia+malaria+LRTI/pneumonia+malnutrition	0	1	0	0	1
anaemia+malaria+LRTI/pneumonia+metabolic acidosis	0	0	1	0	0
anaemia+malaria+LRTI/pneumonia+sepsis	2	0	0	0	2
anaemia+malaria+sepsis	5	4	3	3	9
anaemia+malaria+sepsis+metabolic acidosis	1	0	0	0	1
anaemia+malaria+URTI	0	0	0	1	0
anaemia+convulsions+fever+vomiting	1	0	0	0	1
anaemia+diarrhoea	1	0	1	1	1
anaemia+DUS+sepsis	0	0	0	1	0
anaemia+DUS+sepsis+gastritis	0	0	0	1	0
anaemia+fever	4	3	2	1	7
anaemia+fever+DUS	2	5	1	1	7
anaemia+fever+DUS+malaise	0	0	0	1	0
anaemia+fever+DUS+vomiting	0	1	0	0	1
anaemia+fever+headache	0	0	0	1	0
anaemia+fever+jaundice	0	1	0	0	1
anaemia+fever+other lung condition	0	0	1	0	0
anaemia+fever+shock	0	0	1	0	0
anaemia+gastroenteritis	1	0	1	3	1
anaemia+gastroenteritis+LRTI/pneumonia+phlebitis	0	0	0	1	0
anaemia+gastroenteritis+marasmus	0	0	0	1	0
anaemia+gastroenteritis+sepsis	0	0	0	1	0
anaemia+gastroenteritis+sickle cell crisis	0	1	0	0	1
anaemia+LRTI/pneumonia	5	6	9	1	11
anaemia+LRTI/pneumonia+arthralgia	0	0	0	1	0
anaemia+LRTI/pneumonia+DUS	0	1	0	0	1
anaemia+LRTI/pneumonia+sepsis	2	0	3	1	2
anaemia+lymphadenitis	1	0	0	0	1
anaemia+sepsis	30	19	28	29	49
anaemia+sepsis+cerebral vascular accident	1	0	0	0	1
anaemia+sepsis+convulsions	0	0	0	1	0
anaemia+sepsis+convulsions+sickle cell crisis	0	0	0	1	0
anaemia+sepsis+diarrhoea	0	1	0	0	1
anaemia+sepsis+diarrhoea+sickle cell crisis	1	0	0	0	1
anaemia+sepsis+lymphoma	0	0	1	0	0
anaemia+sepsis+malnutrition	0	0	1	0	0
anaemia+sepsis+shock+disseminated intravascular coagulation	0	1	0	0	1
anaemia+sepsis+sickle cell crisis	3	0	0	1	3

	MVMM and Cotrimoxazole (N=327)	MVMM only (N=343)	Iron and folate and Cotrimoxazole (N=346)	Iron and folate (N=335)	Total (N=670)
anaemia+sepsis+ulcers	0	1	0	0	1
anaemia+sepsis+URTI	0	1	1	0	1
anaemia+TB	0	1	0	0	1
anaemia+vomiting+other lung condition	0	1	0	0	1
anaemia+malaria+sepsis+sickle cell crisis	0	0	1	0	0
malaria	22	33	23	29	55
malaria+convulsions	0	1	0	1	1
malaria+convulsions+epistaxis	0	0	1	0	0
malaria+DUS	4	7	4	6	11
malaria+hypoglycaemia	0	0	0	1	0
malaria+malnutrition	0	1	0	0	1
malaria+sickle cell crisis	1	0	0	1	1
malaria+diarrhoea	1	0	0	0	1
malaria+fever+DUS+convulsions	0	0	0	1	0
malaria+LRTI/pneumonia	4	2	0	0	6
malaria+LRTI/pneumonia+hypotension	0	0	1	0	0
malaria+LRTI/pneumonia+metabolic acidosis+shock	0	1	0	0	1
malaria+LRTI/pneumonia+sepsis	1	0	0	0	1
malaria+LRTI/pneumonia+sepsis+convulsions	1	0	0	0	1
malaria+sepsis	3	2	3	3	5
malaria+URTI+metabolic acidosis	0	1	0	0	1
abscess+osteomyelitis	1	0	0	0	1
abscess+osteomyelitis+sickle cell crisis	1	0	0	0	1
abscess+sickle cell crisis	0	2	0	1	2
acute hepatitis	0	0	0	1	0
bronchiolitis+diarrhoea	1	0	0	0	1
cardiac failure+pneumonia	1	0	0	0	1
cellulitis	0	1	0	0	1
convulsions+fever	2	0	1	0	2
convulsions+fever+diarrhoea	0	0	1	0	0
convulsions+fever+vomiting	0	1	0	1	1
diarrhoea	2	1	1	1	3
diarrhoea+thrombotic thrombocytopenic purpura	0	0	0	1	0
diarrhoea+vomiting	1	0	0	0	1
diarrhoea+vomiting+DUS	0	1	0	0	1
disseminated intravascular coagulation+gingivitis+malnutrition+thrombotic thrombocytopenic purpura	0	0	1	0	0
DUS	4	8	3	4	12
DUS+abdominal pain	0	0	1	0	0
DUS+sickle cell crisis	0	0	0	1	0
DUS+diarrhoea+fever	0	0	0	1	0

	MVMM and Cotrimoxazole (N=327)	MVMM only (N=343)	Iron and folate and Cotrimoxazole (N=346)	Iron and folate (N=335)	Total (N=670)
DUS+fever	1	2	1	2	3
DUS+fever+mouth ulcers	0	0	1	0	0
encephalitis+LRTI/pneumonia	0	0	1	0	0
fever	1	3	4	3	4
fever+abdominal pain	0	1	0	0	1
fever+fatigue	0	2	0	0	2
fever+marasmus	0	2	0	0	2
fever+shortness of breath	0	0	1	1	0
fever+vomiting+fatigue	0	0	1	0	0
fever+vomiting+jaundice	0	0	0	1	0
gastroenteritis	0	2	0	0	2
gastroenteritis+fever+malnutrition	1	0	0	0	1
gastroenteritis+marasmus	0	0	1	0	0
gastroenteritis+measles	0	0	0	1	0
gastroenteritis+urti	0	0	1	0	0
laryngitis+tracheitis	0	1	0	0	1
LRTI/pneumonia	2	8	7	4	10
LRTI/pneumonia+convulsions	0	0	1	0	0
LRTI/pneumonia+marasmus	0	0	0	1	0
LRTI/pneumonia+sepsis	1	0	0	3	1
LRTI/pneumonia+sepsis+gastroenteritis+hypoglycaemia	0	0	0	1	0
LRTI/pneumonia+sepsis+sickle cell crisis	0	0	0	1	0
LRTI/pneumonia+sickle cell crisis	0	1	0	1	1
LRTI/pneumonia+vomiting	0	0	0	1	0
meningitis	0	0	2	0	0
neuroplasticity+metastatis+meningitis	0	0	1	0	0
pharynigitis	1	0	0	0	1
sepsis	9	9	7	7	18
sepsis+diarrhoea	0	0	0	1	0
sepsis+DUS	0	0	1	0	0
sepsis+jaundice	1	0	0	0	1
sepsis+leukaemia	1	0	0	0	1
sepsis+osteomyelitis	1	0	0	0	1
sepsis+sickle cell crisis	1	3	5	2	4
sepsis+vomiting	0	0	0	1	0
septic arthritis+sickle cell crisis	0	1	0	0	1
TB	0	0	1	0	0
TB (abdominal)+malnutrition	0	0	0	1	0
TB (disseminated)	0	0	0	1	0
TB (pulmonary)	0	1	1	0	1
urinary tract infection	0	0	0	2	0

	MVMM and Cotrimoxazole (N=327)	MVMM only (N=343)	Iron and folate and Cotrimoxazole (N=346)	Iron and folate (N=335)	Total (N=670)
allergic reaction	11	5	6	7	16
allergic reaction+hypotension	0	1	0	0	1
abdominal pain	1	0	0	0	1
abdominal pain+vomiting	1	0	0	0	1
arthritis	0	1	0	0	1
bone marrow failure+leukaemia+petechia	0	1	0	0	1
brochospasm+asthma	0	1	0	0	1
bronchiolitis	0	0	0	1	0
burns	1	1	0	0	2
carcinoma	0	0	1	0	0
cardiac failure	1	0	0	0	1
chronic renal failure	0	1	0	0	1
convulsions	0	1	0	0	1
cortical blindness	0	1	0	0	1
cough+shortness of breath	1	0	0	0	1
epistaxis+cough	0	1	0	0	1
fracture	0	1	0	1	1
haemolysis	0	0	3	0	0
hepatic failure+jaundice	0	0	1	0	0
hernia	0	1	0	0	1
hypotension	2	1	2	2	3
impetigo	0	2	0	0	2
injury	0	0	0	1	0
intestinal obstruction	0	0	1	0	0
jaundice	0	1	0	0	1
kwashiorkor	0	0	0	1	0
leukaemia	6	2	2	1	8
leukaemia+chicken pox	0	1	0	0	1
leukaemia+thrombocytopenia	1	0	0	0	1
malnutrition	0	1	1	2	1
malnutrition+sickle cell crisis	0	0	0	1	0
marasmus	0	0	0	1	0
neuroblastoma	1	0	1	0	1
neuroplasticity+metastatis	0	0	1	0	0
non-hodgkins lymphoma	0	0	2	0	0
oedema	1	0	0	0	1
oesophageal reflux	0	0	1	0	0
pancytopenia+splenomegaly	1	0	0	0	1
pulmonary oedemia	0	0	0	1	0
respiratory failure	1	2	2	0	3
respiratory failure+hypotension+pleural effusion	0	0	1	0	0

	MVMM and Cotrimoxazole (N=327)	MVMM only (N=343)	Iron and folate and Cotrimoxazole (N=346)	Iron and folate (N=335)	Total (N=670)
shock	0	0	0	1	0
shortness of breath	0	1	0	0	1
sickle cell crisis	4	8	9	5	12
sickle cell crisis+fatigue	1	0	0	0	1
splenic cysts	0	1	0	0	1
stroke/cerebrovascular accident	0	1	1	0	1
thromobocytopenia	0	0	0	1	0
transfusion overload	0	1	0	0	1
urticaria	4	3	4	0	7
vomiting	0	1	0	0	1

Table S5 Heterogeneity tests between different factorial randomisations for primary and secondary time-to-event outcomes

p-values for heterogeneity	MVMM vs cotrimoxazole	MVMM vs 30 vs 20 ml/kg	MVMM vs immediate vs no immediate transfusion	Cotrimoxazole vs 30 vs 20 ml/kg	Cotrimoxazole vs immediate vs no immediate transfusion
28 day mortality	0.74	0.74	0.24	0.12	0.65
90 day mortality	0.52	0.35	0.77	0.44	0.74
Development of severe anaemia post discharge	0.38	0.71	0.88	0.22	0.48
Readmission to hospital	0.23	0.86	0.08	0.73	0.89
Any serious adverse event	0.09	0.62	0.15	0.29	0.89

Table S6 Grade 3 or 4 toxicity adverse events that were definitely, probably or possibly related as assigned by the clinician

**Clinician assigned relatedness**	**Cotrimoxazole**	**No cotrimoxazole**	**Total**
Definitely	0	0	0
Probably	3<br>(hypersensitivity reaction; anaemia; anaemia)	0	3
Possibly	7<br>(diarrhoea; anaemia+sepsis+sickle cell anaemia; urticaria; dark urine syndrome; anaemia+malaria; anaemia; jaundice+sepsis)	0	7
**Clinician assigned relatedness**	**MVMM**	**Iron and folate**	**Total**
Definitely	0	0	0
Probably	1<br>(anaemia)	0	1
Possibly	3<br>(Anaemia; vomiting; acute diarrhoea)	1<br>(dark urine syndrome)	4

Note: All events had independent clinical review and none were adjudicated to be probably or possibly related to the interventions.

Table S7 Secondary and other clinical outcomes within secondary population

(a) MVMM randomisation	MVMM N participants (% of 1913)	Iron/folate N participants (% of 1889)	Total N participants (% of 3802)	Hazard Ratio (95% CI)	p
Death					
- 28 days*	26 (1%)	32 (2%)	58 (2%)	0.81 (0.48-1.35)	0.41
- 90 days*	75 (4%)	75 (4%)	150 (4%)	0.99 (0.72-1.37)	0.96
- 180 days (primary outcome)	122 (6%)	121 (6%)	243 (6%)	1.00 (0.76-1.28)	0.98
Development of severe anaemia (Hb<6g/dl) post discharge*	382 (20%)	383 (20%)	765 (20%)	0.98 (0.85-1.13)	0.81
Readmission to hospital*	330 (17%)	342 (18%)	672 (18%)	0.95 (0.82-1.10)	0.49
Any serious adverse event* [number of events]	439 (23%) [616]	450 (24%) [615]	889 (23%) [1231]	0.96 (0.84-1.09)	0.54
Any anaemia SAE [number of events]	251 (13%) [342]	262 (14%) [352]	513 (13%) [694]	-	0.51
Any malaria SAE [number of events]	134 (7%) [153]	132 (7%) [149]	266 (7%) [302]	-	1.00
Any sepsis SAE [number of events]	76 (4%) [98]	88 (5%) [104]	164 (4%) [202]	-	0.30
Any haemoglobinuria SAE [number of events]	54 (3%) [66]	56 (3%) [64]	110 (3%) [130]	-	0.85
**(b) Cotrimoxazole randomisation**	**Cotrimoxazole N particpants (% of 1908)**	**No cotrimoxazole N participants (% of 1921)**	**Total N participants (% of 3829)**	**Hazard Ratio (95% CI)**	**p**
Death					
- 28 days*	36 (2%)	23 (1%)	59 (2%)	1.59 (0.94-2.68)	0.08
- 90 days*	80 (4%)	72 (4%)	152 (4%)	1.13 (0.82-1.55)	0.46
- 180 days (primary outcome)	122 (6%)	123 (6%)	245 (6%)	1.00 (0.78-1.29)	0.95

(a) MVMM randomisation	MVMM<br>N participants<br>(% of 1913)	Iron/folate<br>N participants<br>(% of 1889)	Total<br>N participants<br>(% of 3802)	Hazard Ratio<br>(95% CI)	p
Development of severe anaemia (Hb<6g/dl) post discharge*	397 (21%)	369 (19%)	766 (20%)	1.11 (0.96-1.28)	0.15
Readmission to hospital*	324 (17%)	346 (18%)	670 (18%)	0.94 (0.81-1.09)	0.42
Any serious adverse event* [number of events]	438 (23%)[596]	451 (23%)[625]	889 (23%)[1221]	0.98 (0.86-1.12)	0.78
Any anaemia SAE [number of events]	258 (13%)[343]	242 (13%) [333]	500 (13%) [676]	-	0.42
Any malaria SAE [number of events]	116 (6%)[130]	150 (8%)[172]	266 (7%)[302]	-	0.04
Any sepsis SAE [number of events]	92 (5%) [107]	71 (4%)[96]	163 (4%)[203]	-	0.09
Any haemoglobinuria SAE [number of events]	50 (3%) [58]	60 (3%)[72]	110 (3%) [130]	-	0.38

* secondary outcome prespecified in the protocol

† estimated from competing risks sub-hazard regression.

[&] Fisher's Exact test

Note: secondary population is patients alive at the minimum of discharge or 5 days from randomisation in whom interventions were neither mandated nor contraindicated

Table S8 Changes in weight and MUAC within the secondary population

(a) MVMM randomisation	MVMM Mean (95% CI)	Iron/folate Mean (95% CI)	Difference Mean (95% CI) *
**90 days**			
Change in weight from baseline (kg)	1.24 (1.19,1.31); N=1729	1.19 (1.12,1.25); N=1712	0.06 (-0.03,0.15)
Change in MUAC from baseline (cm)	0.44 (0.40,0.48); N=1734	0.43 (0.39,0.48); N=1714	0.01 (-0.04,0.07)
**180 days**			
Change in weight from baseline (kg)	1.81 (1.75,1.88); N=1646	1.78 (1.71,1.85); N=1636	0.03 (-0.07,0.12)
Change in MUAC from baseline (cm)	0.58 (0.54, 0.63); N=1662	0.62 (0.57,0.66); N=1647	-0.02 (-0.08,0.04)
**(b) Cotrimoxazole randomisation**	**Cotrimoxazole Mean (95% CI)**	**No cotrimoxazole Mean (95% CI)**	**Difference Mean (95% CI) ***
**90 days**			
Change in weight from baseline (kg)	1.25 (1.19, 1.31); N=1725	1.18 (1.12,1.24); N=1737	0.06 (-0.02,0.15)
Change in MUAC from baseline (cm)	0.45 (0.41, 0.50); N=1725	0.48 (0.43,0.52); N=1745	0.00 (-0.06,0.06)
**180 days**			
Change in weight from baseline (kg)	1.83 (1.76, 1.90); N=1640	1.77 (1.71, 1.84); N=1661	0.06 (-0.04,0.15)
Change in MUAC from baseline (cm)	0.62 (0.56,0.67); N=1656	0.66 (0.61, 0.71); N=1676	-0.02 (-0.09,0.04)

* Estimated differences and confidence intervals obtained from a linear regression adjusted for baseline values.

Note: secondary population is patients alive at the minimum of discharge or 5 days from randomisation in whom interventions were neither mandated nor contraindicated.

Table S9 Numbers with haemoglobin <6 and >9 g/dl over time from randomisation

	8 hours	16 hours	24 hours	48 hours	28 days	90 days	180 days
**MVMM randomisation: >9 g/dl**							
N:MVMM	293 (15%)	332 (17%)	330 (17%)	318 (16%)	1032 (57%)	1037 (60%)	1036 (62%)
N:Iron&folate	300 (16%)	349 (18%)	330 (17%)	347 (18%)	1068 (59%)	1064 (62%)	1065 (64%)
p-value*	0.35	0.18	0.60	0.12	0.25	0.20	0.32
**MVMM randomisation: <6 g/dl**							
N:MVMM	603 (31%)	563 (29%)	523 (27%)	485 (25%)	142 (8%)	132 (8%)	124 (7%)
N:Iron&folate	620 (32%)	544 (28%)	547 (28%)	510 (26%)	136 (7%)	134 (8%)	136 (8%)
p-value*	0.67	0.44	0.46	0.51	0.60	0.93	0.49
**Cotrimoxazole randomisation: >9 g/dl**							
N:Cotrimoxazole	291 (15%)	338 (17%)	337 (17%)	358 (19%)	1040 (57%)	1053 (61%)	1054 (63%)
N:SoC	302 (16%)	343 (18%)	323 (17%)	307 (16%)	1060 (58%)	1048 (60%)	1047 (62%)
p-value*	0.70	0.94	0.51	0.02	0.49	0.57	0.58
**Cotrimoxazole randomisation: <6 g/dl**							
N: Cotrimoxazole	605 (31%)	553 (28%)	538 (28%)	528 (27%)	142 (8%)	132 (8%)	137 (7%)
N:SoC	618 (32%)	554 (29%)	532 (27%)	467 (24%)	136 (7%)	134 (8%)	123 (7%)
p-value*	0.73	0.99	0.80	0.02	0.69	0.98	0.33
**Number of measurements**							
N measurements	3847	3879	3892	3866	3630	3463	3346
Missing due to death (%)	32 (1%)	50 (1%)	58 (1%)	68 (2%)	129 (3%)	231 (6%)	325 (8%)
Missing due to other reason (%)	104 (3%)	54 (1%)	33 (1%)	49 (1%)	224 (6%)	289 (7%)	312 (8%)

* rate ratio from Poisson regression, adjusting for baseline haemoglobin

Note: SoC=Standard of Care
